# Supplementary material for: Interobserver variability in target definition for stereotactic arrhythmia radioablation
Source: Front Cardiovasc Med. 2023 Sep 20;10:1267800. doi: 10.3389/fcvm.2023.1267800 (PMC10547862; doi:10.3389/fcvm.2023.1267800)
Supplement: Supplementary file 1 [file Datasheet1.pdf]

SUPPLEMENTARY MATERIAL

DELINEATION INSTRUCTIONS

Delineation instructions step 1
Delineation instructions step 2
Delineation instructions step 3

CASE DESCRIPTIONS

Case 1
Case 2
Case 3

DELINEATION INSTRUCTIONS

Delineation instructions step 1

Delineate the **spinal canal based on the bony limits of the vertebra** in **each of the provided CT-scan** slices for all **3 patients**. For this first step of the study use the preset ‘window and level’ setting, please **do not change** these settings. It is recommended to use a mouse instead of trackpad.

Please note: you may notice abnormalities in the CT-scan, for this study these are irrelevant.

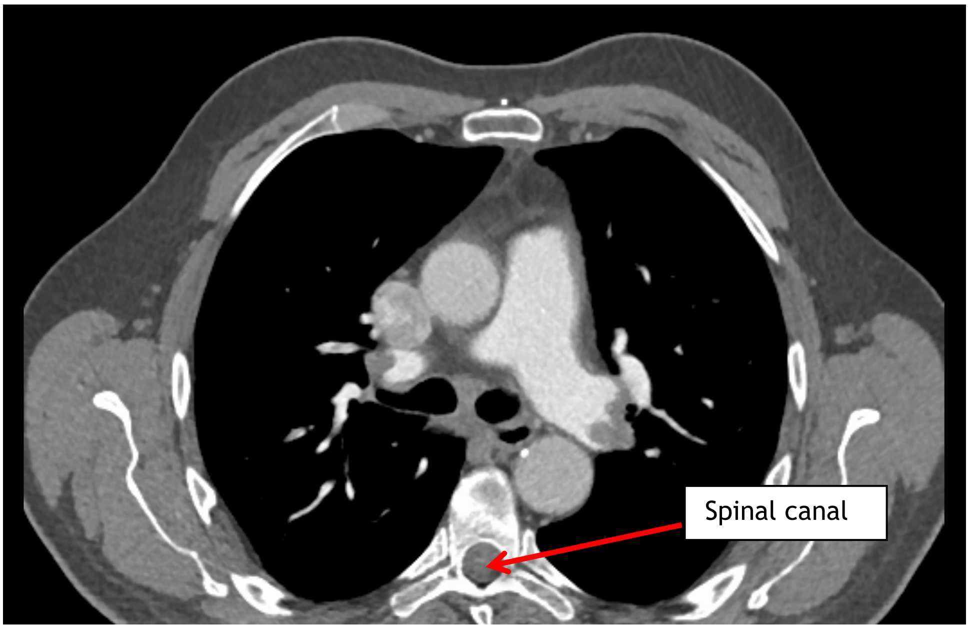

Delineation instructions step 2

Based on the provided case descriptions delineate the Ventricular Tachycardia treatment target for the 3 patient cases: the clinical target volume (CTV). This CTV does not include any additional (uncertainty) margins. The following targeting rules apply:

- **Rule #1: "Choose scar or border zone, not healthy tissue"**
- **Rule #2: "Choose only scar or border zone near VT exit sites, not necessarily the entire scar"**
- **Rule #3: "Choose a single larger area, not multiple small areas"**
- **Rule #4: "Goldilocks principle"**
    - Delineate too small, and you might miss the VT circuit
    - Delineate too large, and there is (likely) higher risk for normal tissue injury

It is recommended to use a mouse instead of trackpad.
Please note: you may notice other abnormalities in the CT-scans,

Delineation instructions step 3

For this final step of the study, we ask you to delineate predefined segments from the [17-segment model](#) (figure) per patient:

- Patient 4: delineate segment **1** – Basal-Anterior
- Patient 5: delineate segment **9** - Mid-Inferoseptal
- Patient 6: delineate segment **16** – Apical-Lateral

It is recommended to use a mouse instead of trackpad.

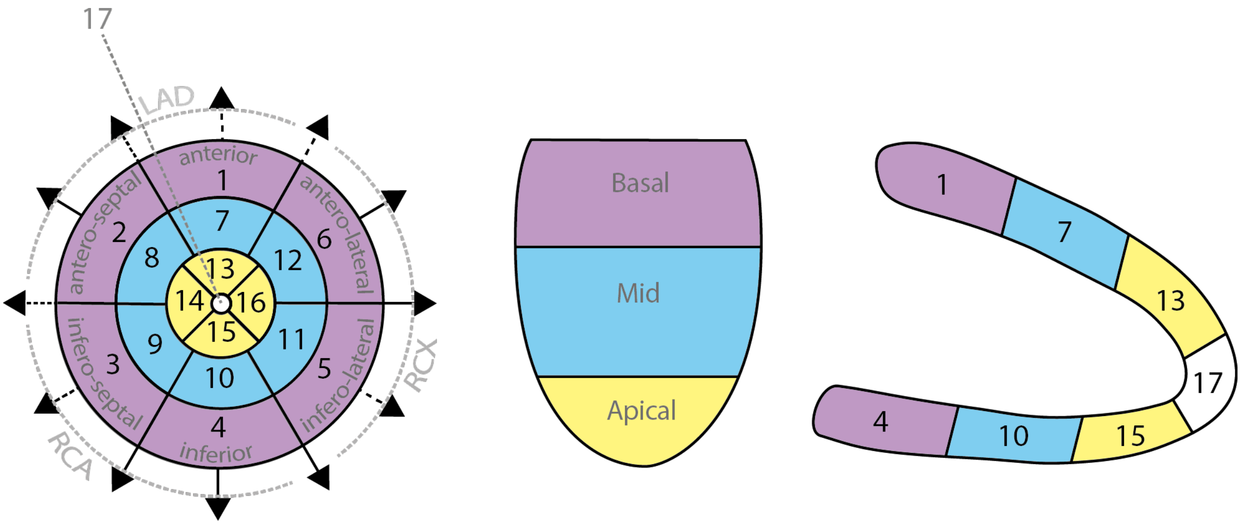

CASE DESCRIPTIONS

Case 1

Abbreviated Clinical History

67 year old with NICM, NYHA class 3 HF, ICD shocks

- 2009: Mitral valve ring + NICM
- 2014: Aborted SCA
- 8/2014: VT ablation #1 (endo)
- 2/2017: VT ablation #2 (endo+surgical epi)
- 1/2018: VT ablation #3 (endo+thoracotomy epi)—2 VTs induced, none clinical, no ablation
- 2/2018: VT ablation #4 (endo)—2 VTs, none clinical, no ablation, required 2 pressors & CPR
    - Impression from operators: thought to be MCV region, but could not pass RF catheter

Abbreviated Medical History

- Antiarrhythmics:
  - Presently on Amio + Mex
  - Previously on Sotalol, Flecainide, Ranolazine
- LVEF recently 50% → 30-35%
- Has MDT Evera with 5076 and 6935M (7/2014). APVS 99%.
- CXR shows an abandoned V lead.

TARGETING DATA FORM

- Electrical Mapping
  - ☒ 12-lead Electrocardiogram (VT exit site)
  - ☒ Noninvasive Electrocardiographic Imaging (VT exit site)
  - ☐ Recent Invasive Catheter Map (activation, pace-map, prior radiofrequency ablation)

- Ventricular Scar Mapping
  - ☒ Echocardiogram (regional wall motion abnormality)
  - ☒ Nuclear Perfusion (non-viability)
  - ☒ PET Scan
  - ☒ Magnetic Resonance (gadolinium enhancement, wall motion abnormality, wall thinning)
  - ☒ Computed Tomography (wall thinning)
  - ☐ Recent Invasive Catheter Map (low amplitude electrograms)

Case: Electrical-12-lead Electrocardiogram (VT1 exit site)

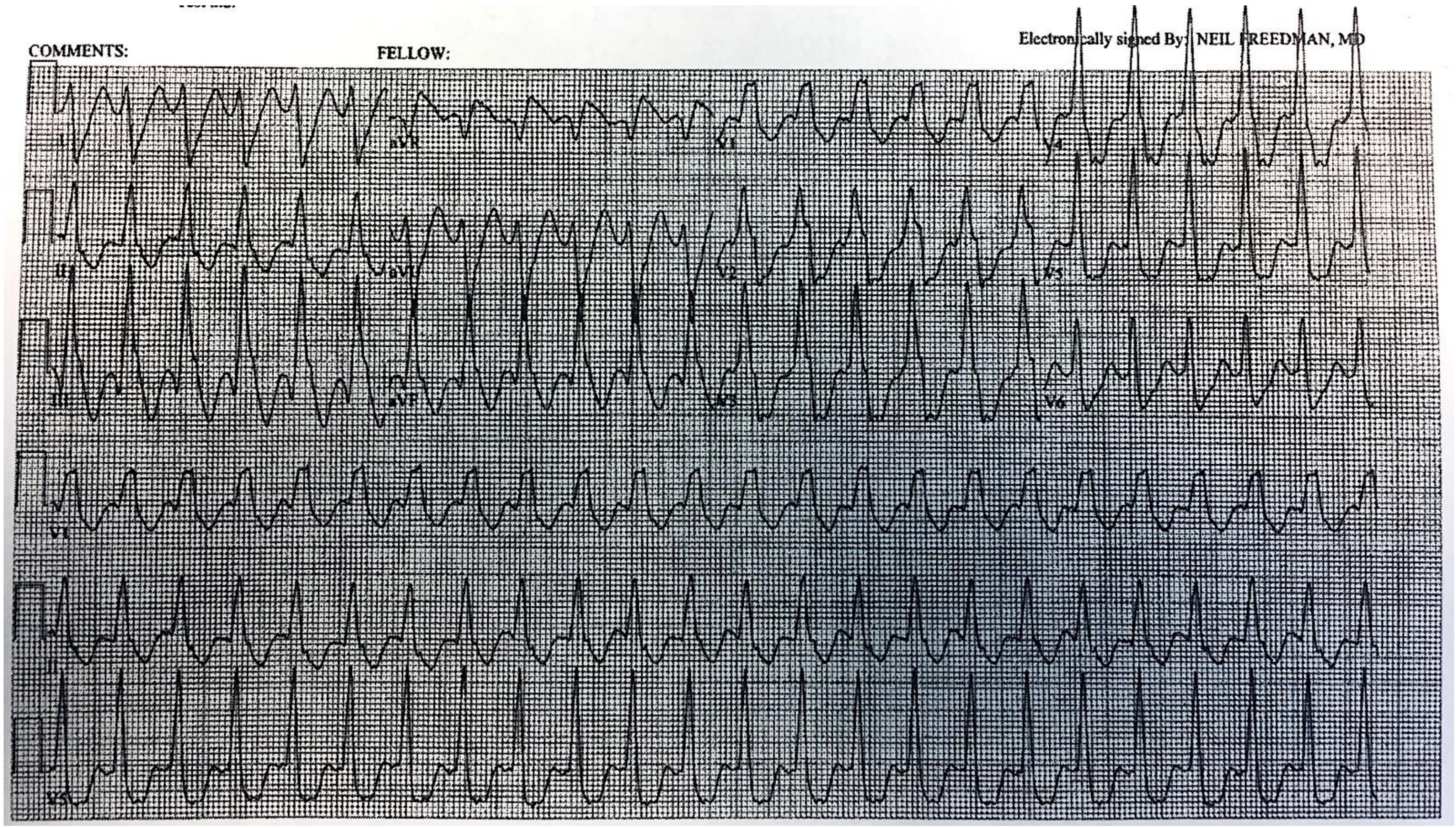

Case: Electrical-12-lead Electrocardiogram (VT2 exit site)

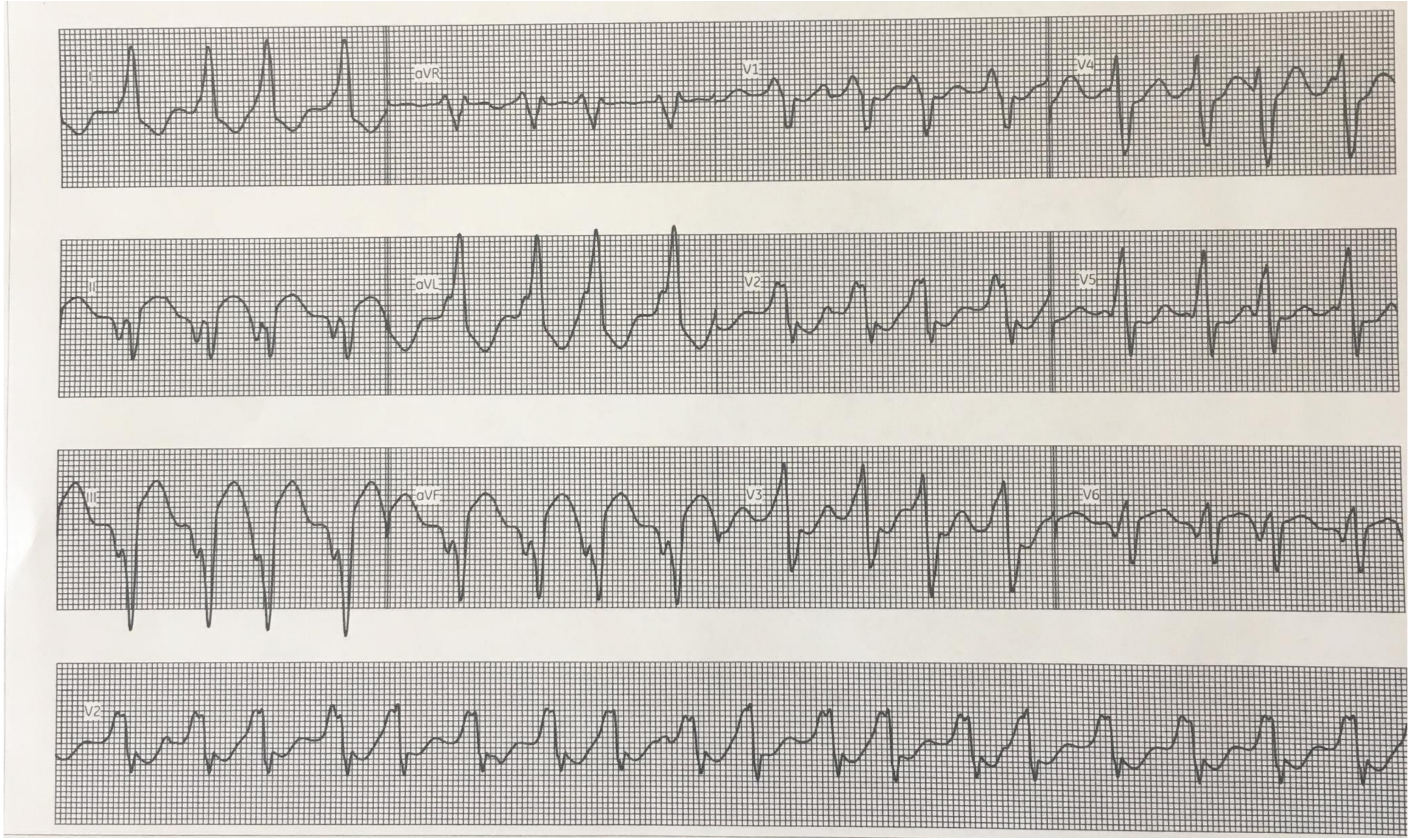

Case: Noninvasive Electrocardiographic Imaging (VT2 exit site)

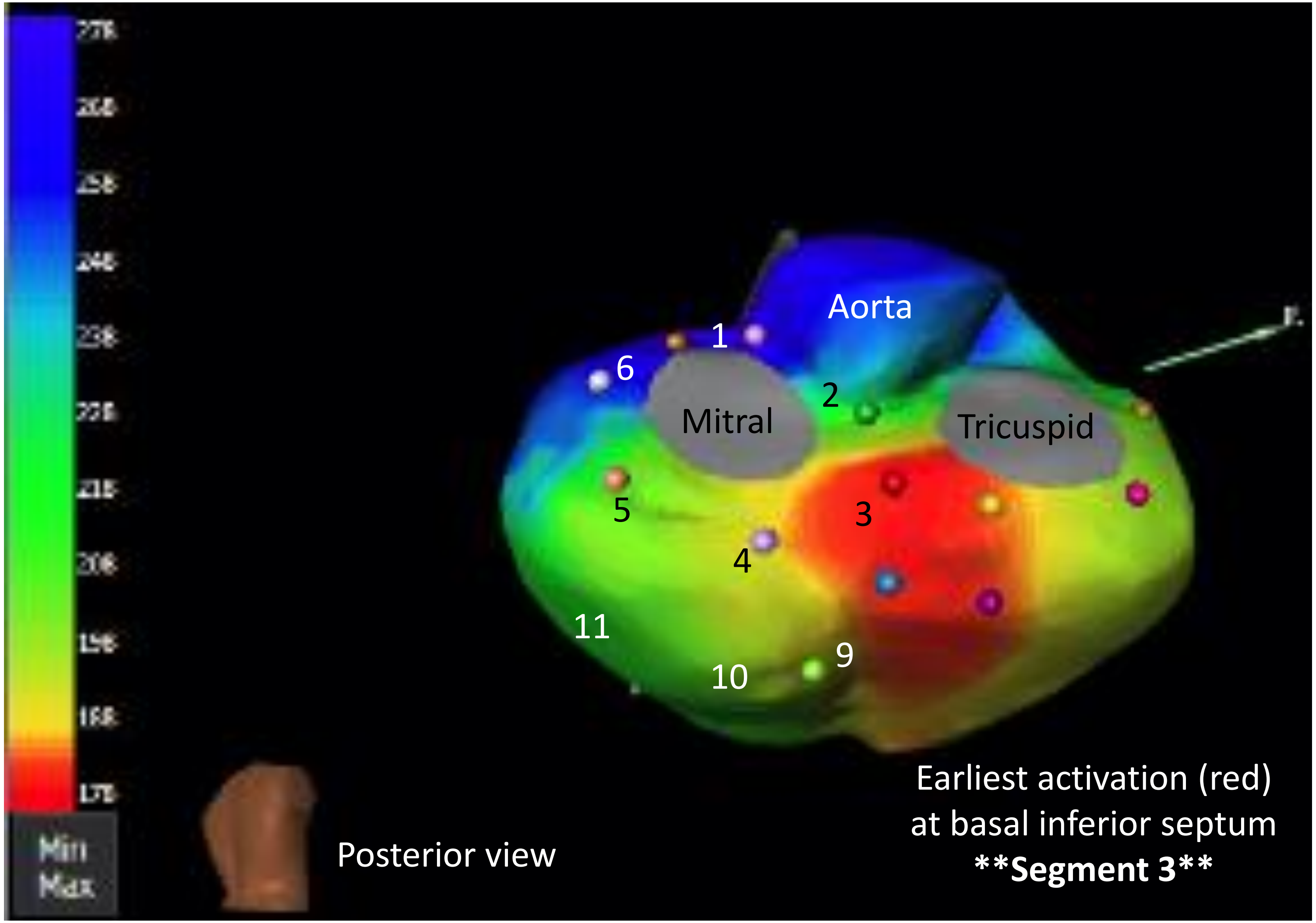

Case: Scar Map -Nuclear Perfusion

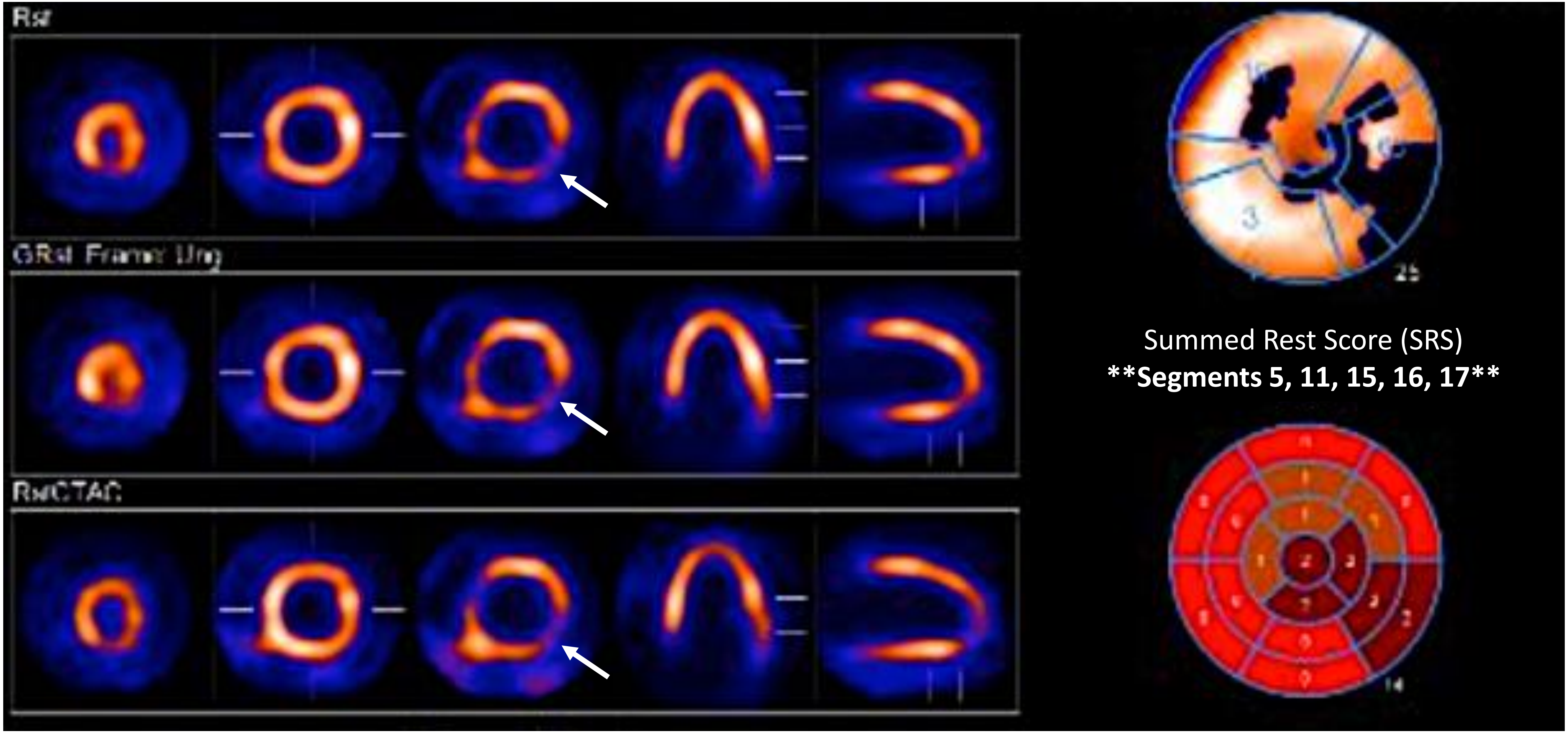

<u>Case:</u> Scar Map-Myocardial Resonance (MRI)

Basal segments: aorta, aortic valve, LV outflow, mitral valve

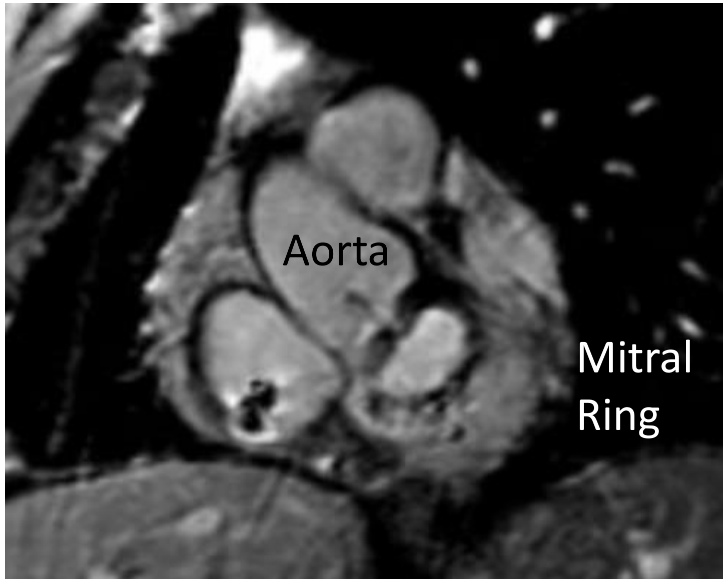

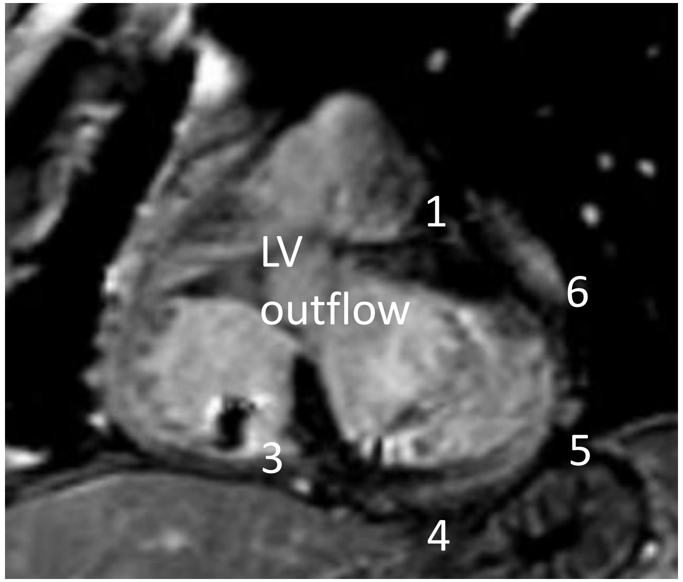

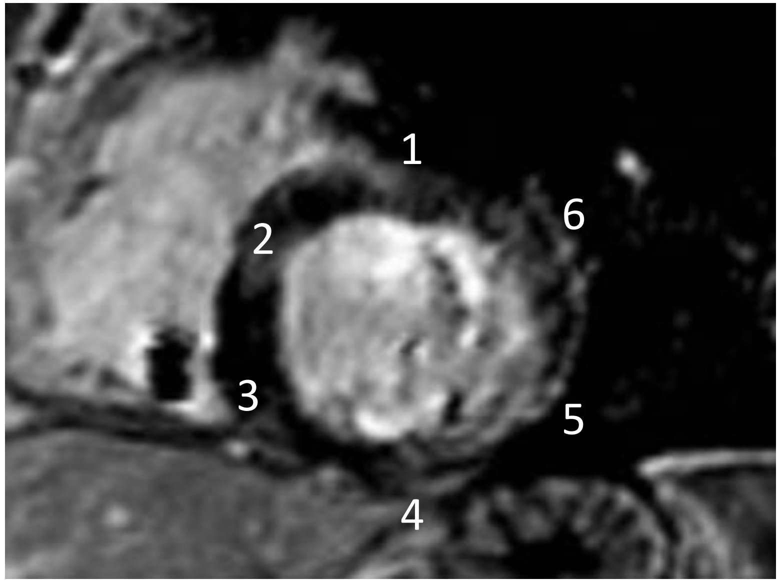

Gadolinium enhancement
****Segments 4 & 5****

<u>Case:</u> Scar Map-Myocardial Resonance (MRI)

Mid segments: papillary muscles

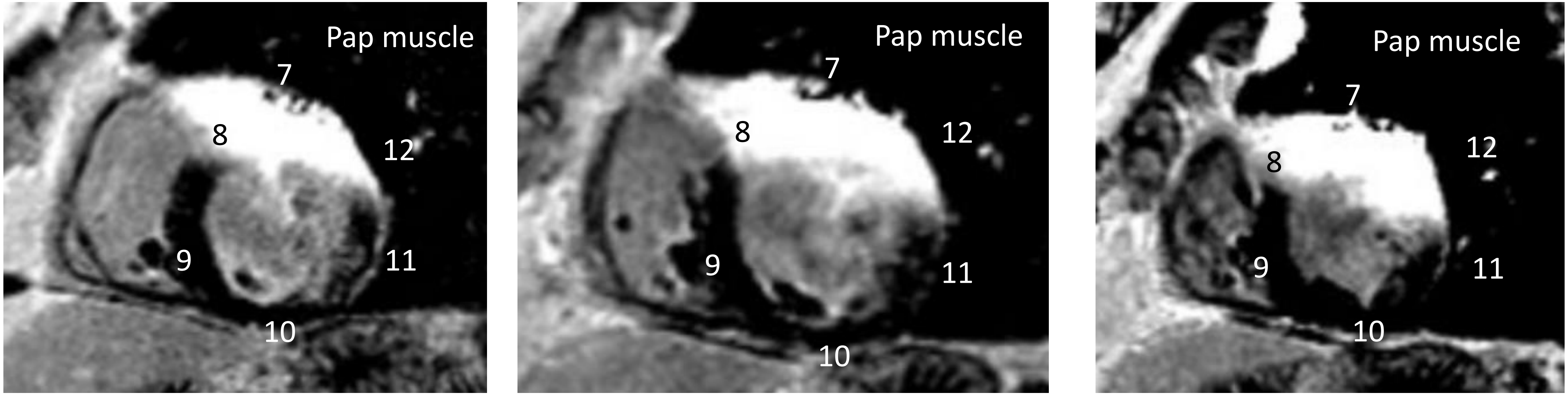

Gadolinium enhancement
Segment 11

Not interpretable:
Segments 7, 8, 12

Case: Scar Map-Myocardial Resonance (MRI)

Apical segments: no papillary muscle

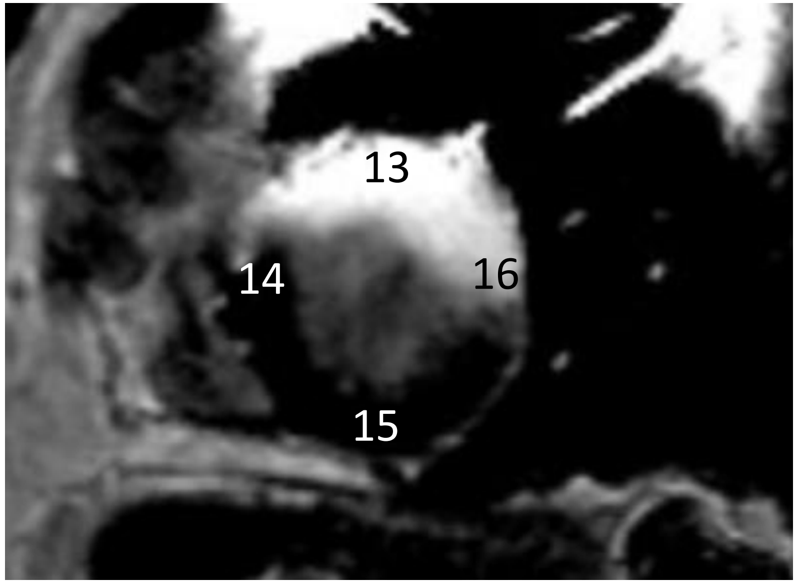

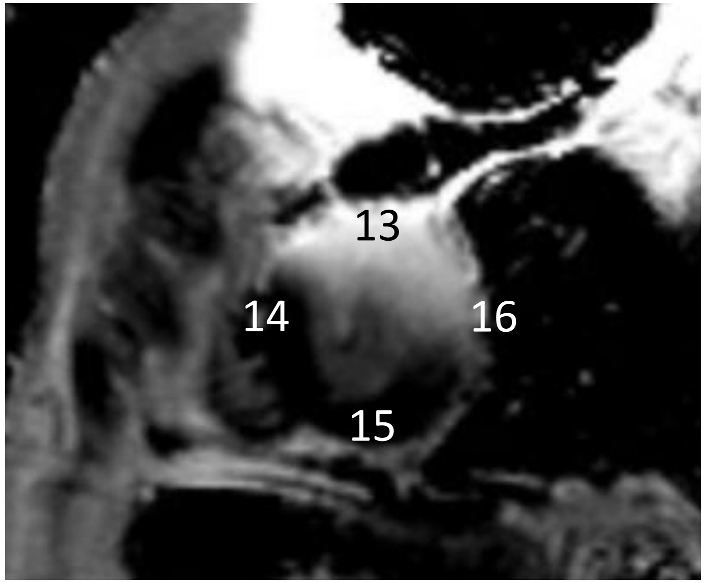

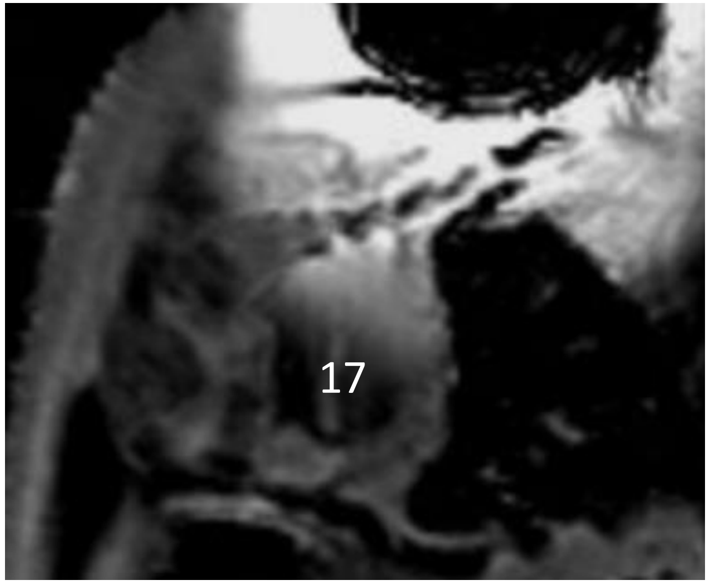

Gadolinium enhancement
****None****

Not interpretable:
Segment 13, 16

Case: Scar Map-Myocardial Resonance (MRI), long axis

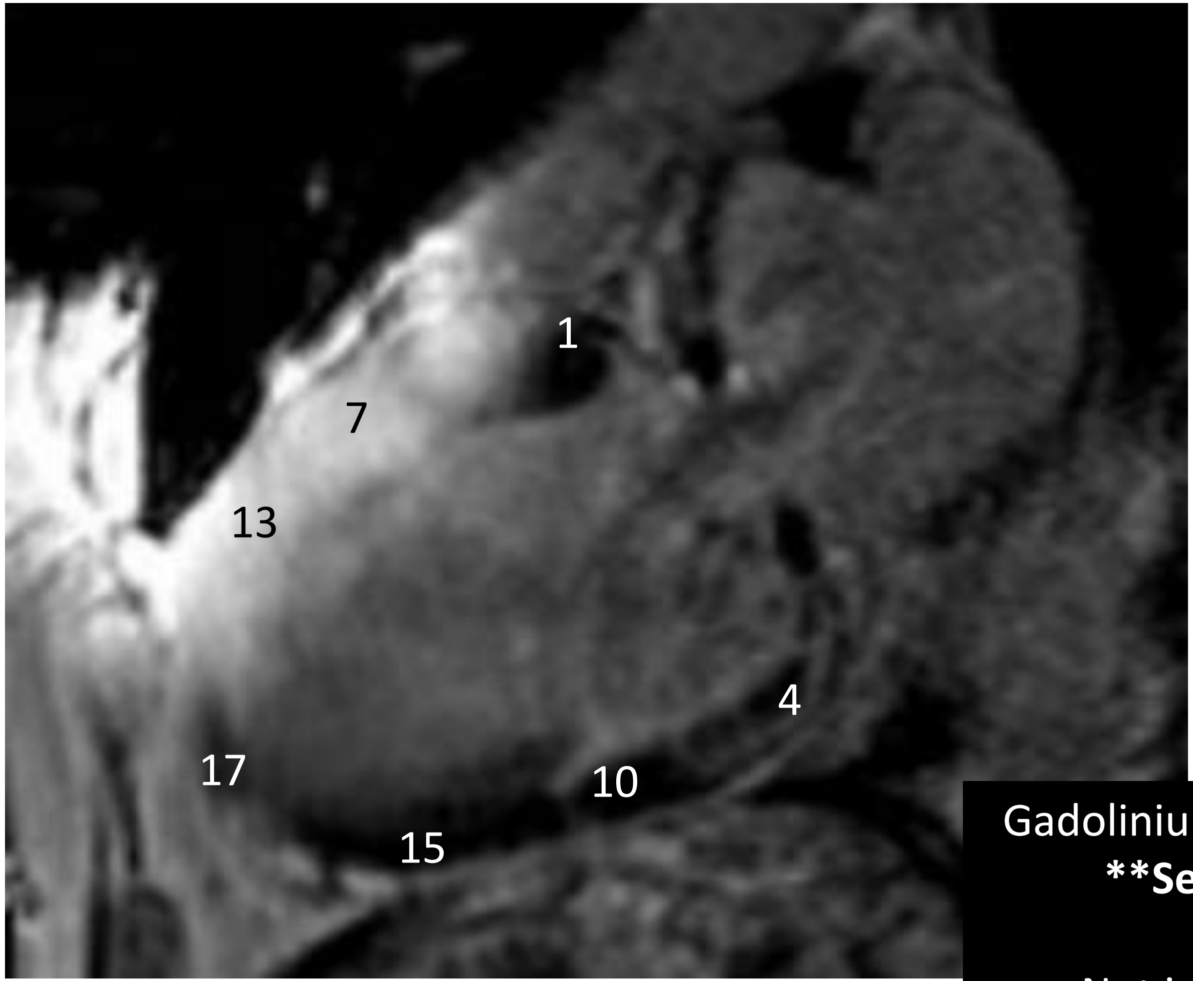

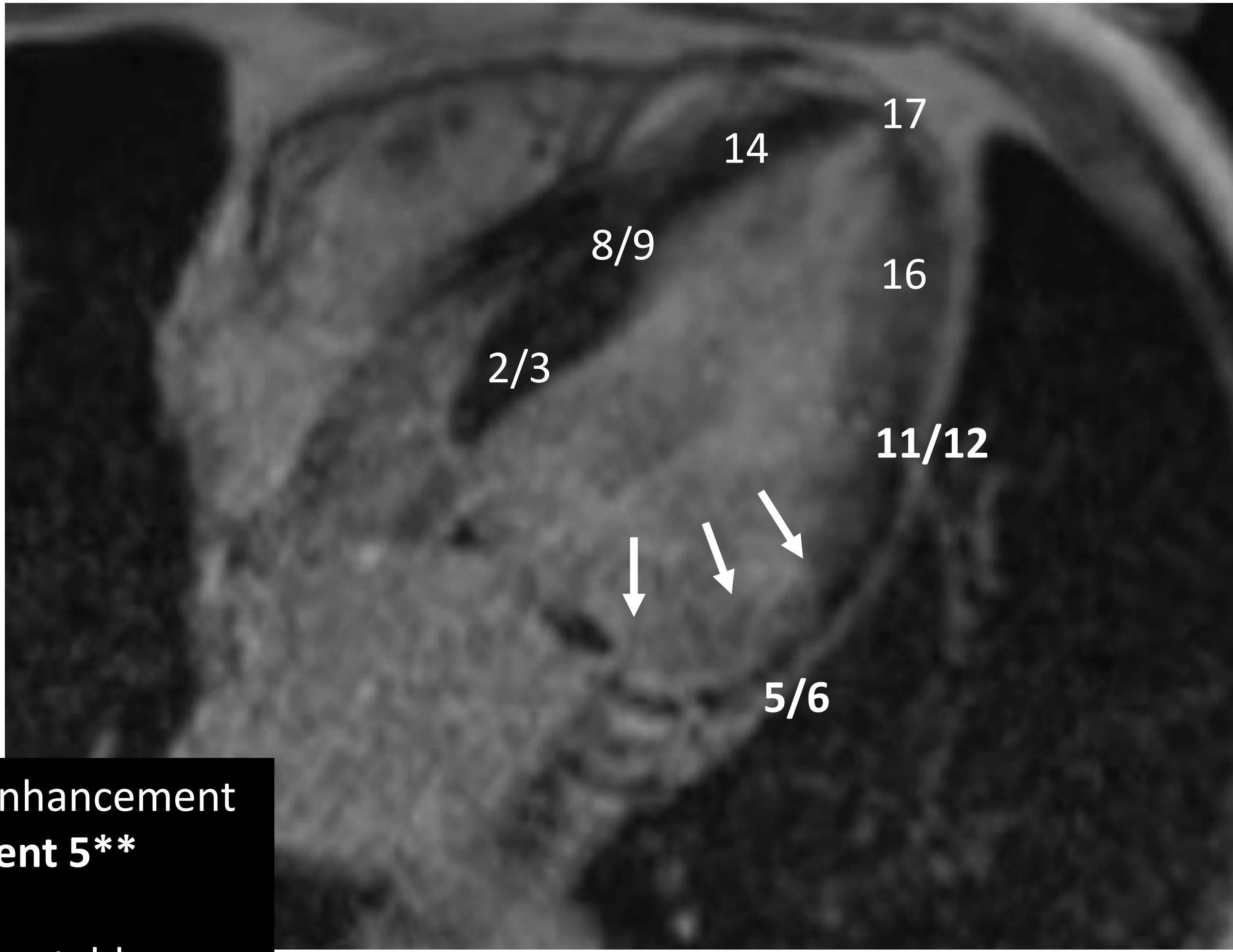

Gadolinium enhancement
Segment 5

Not interpretable:
Segments 7, 13

<u>Case:</u> Scar Map-Myocardial Resonance (MRI)

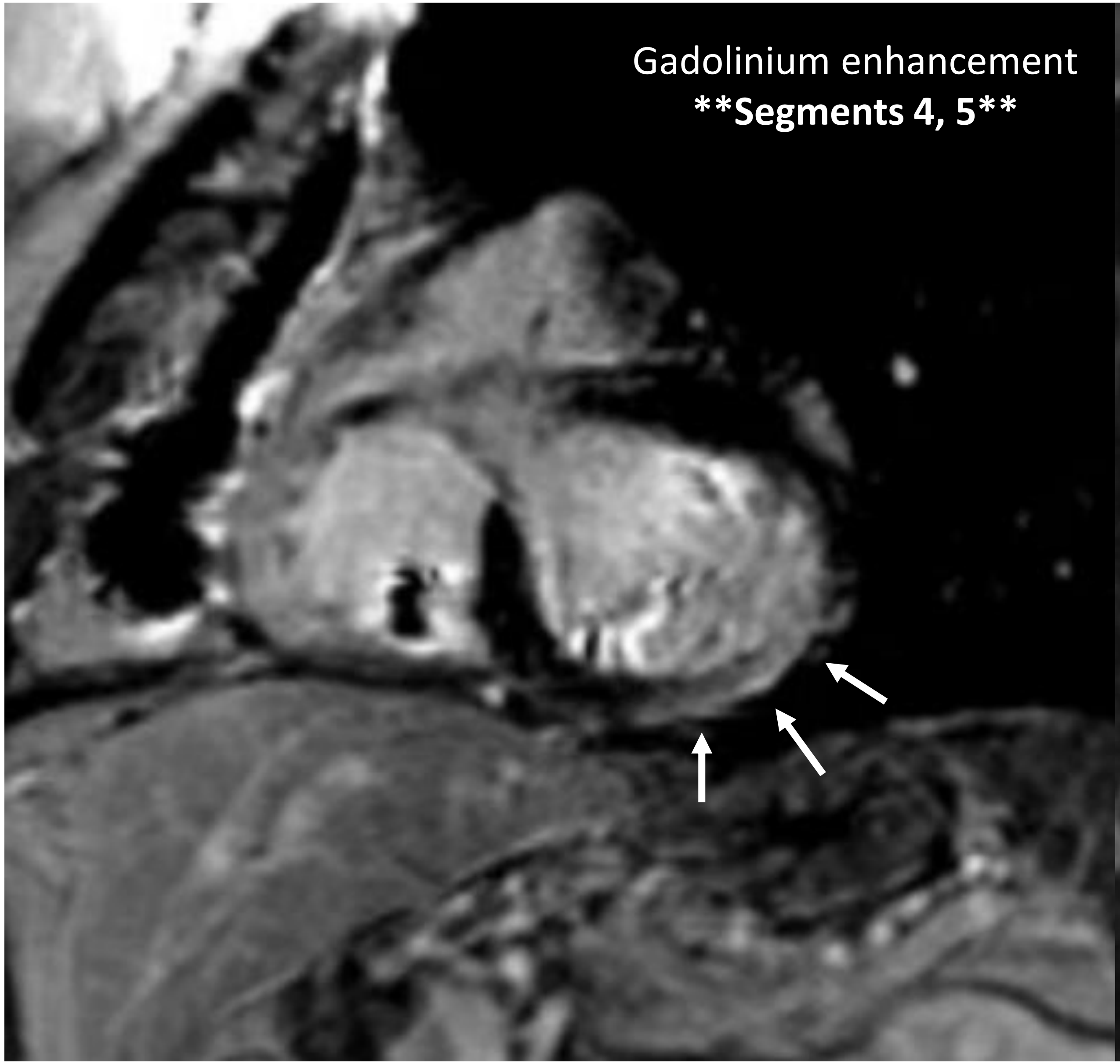

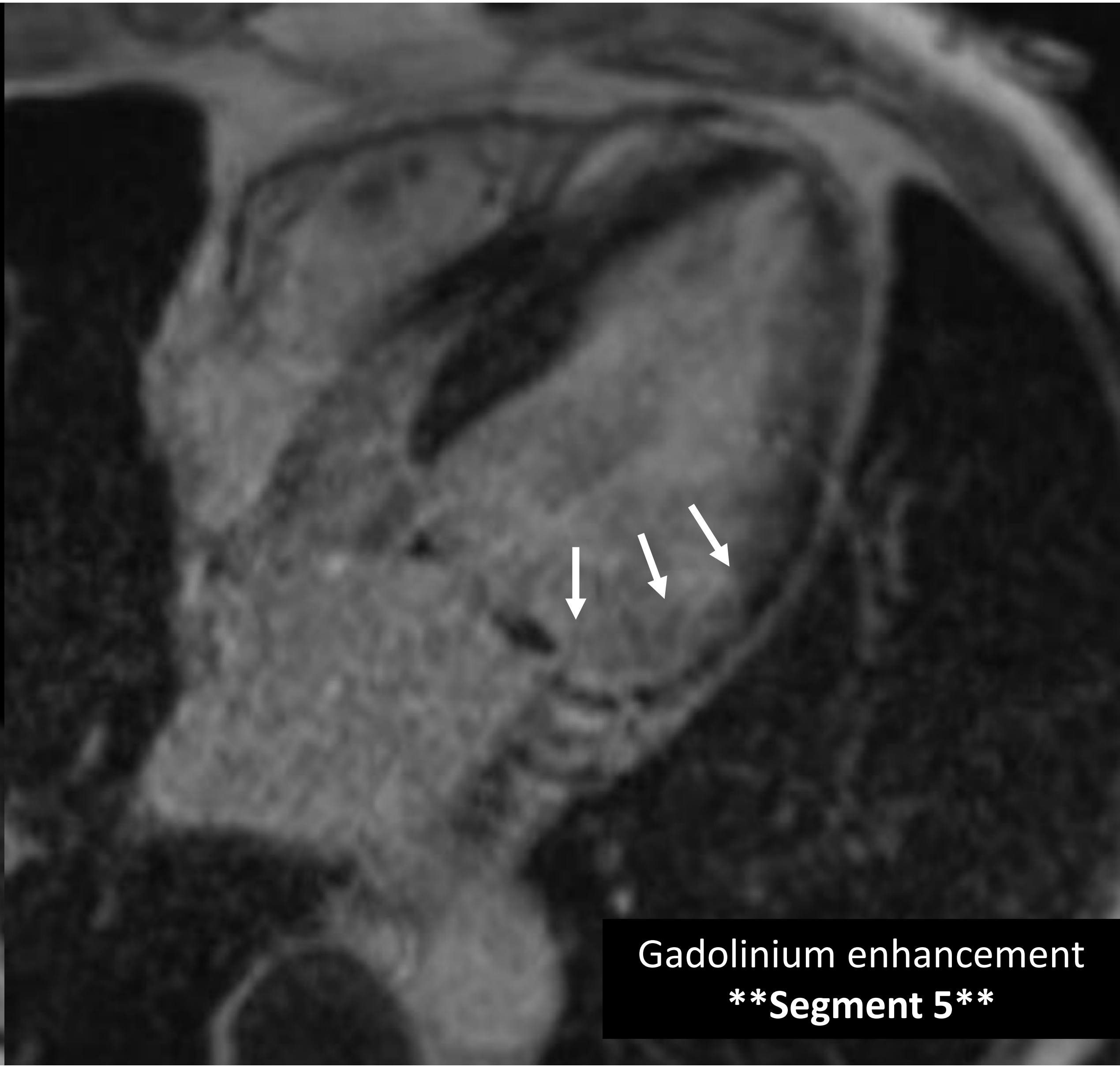

<u>Case:</u> Scar Map-Computed Tomography (CT)

Superior | Superior-Mid | Inferior-Mid | Inferior

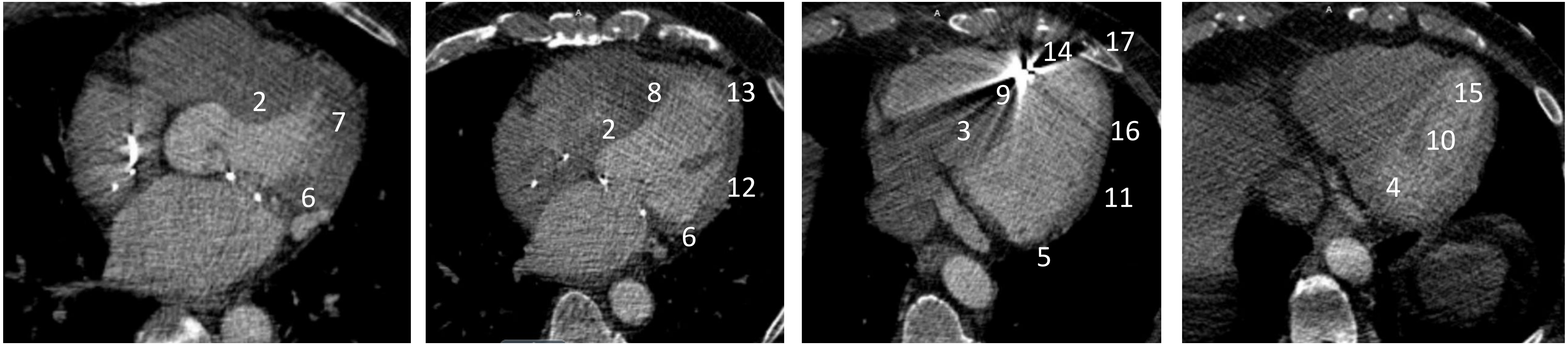

Wall thinning
****Segment 5****

<u>Case:</u> Scar Map-Echo Wall Motion Abnormality

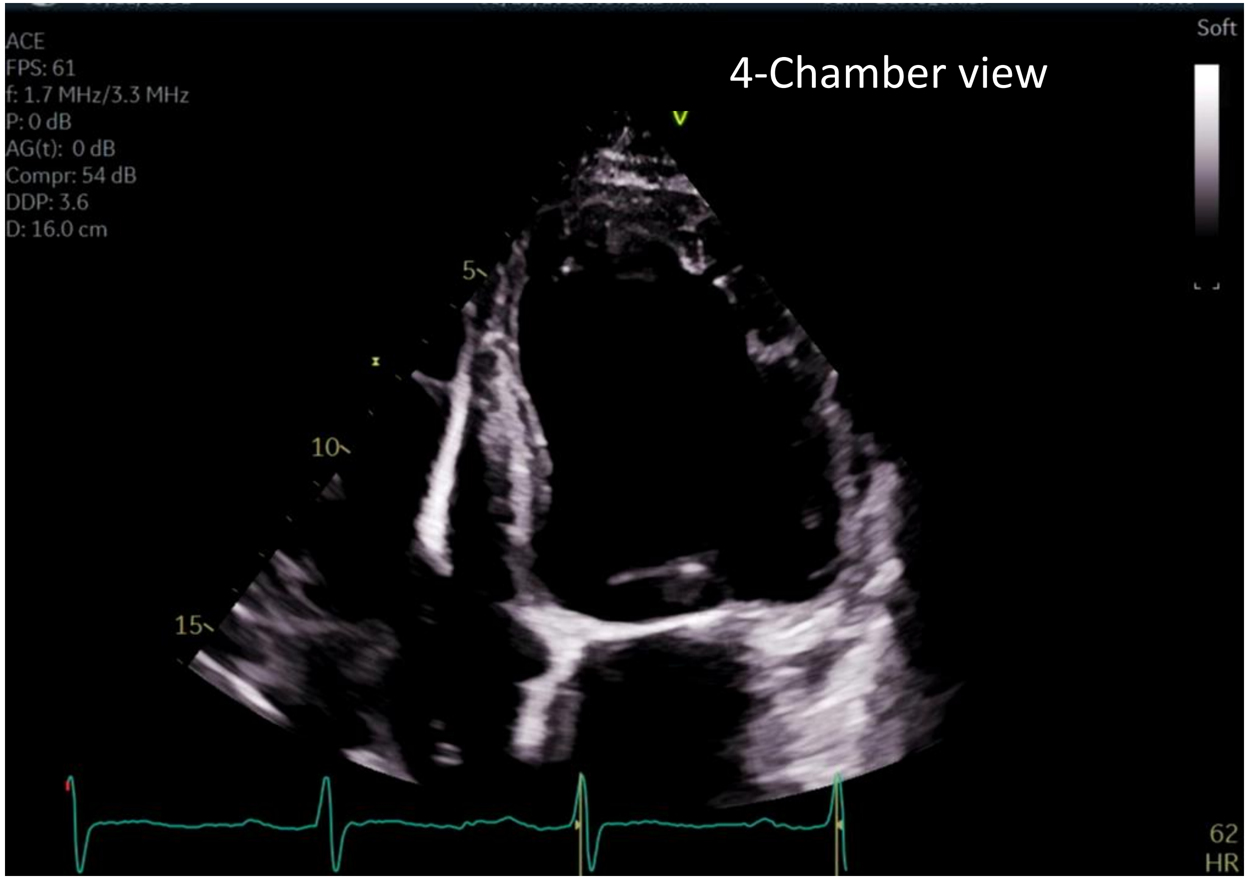

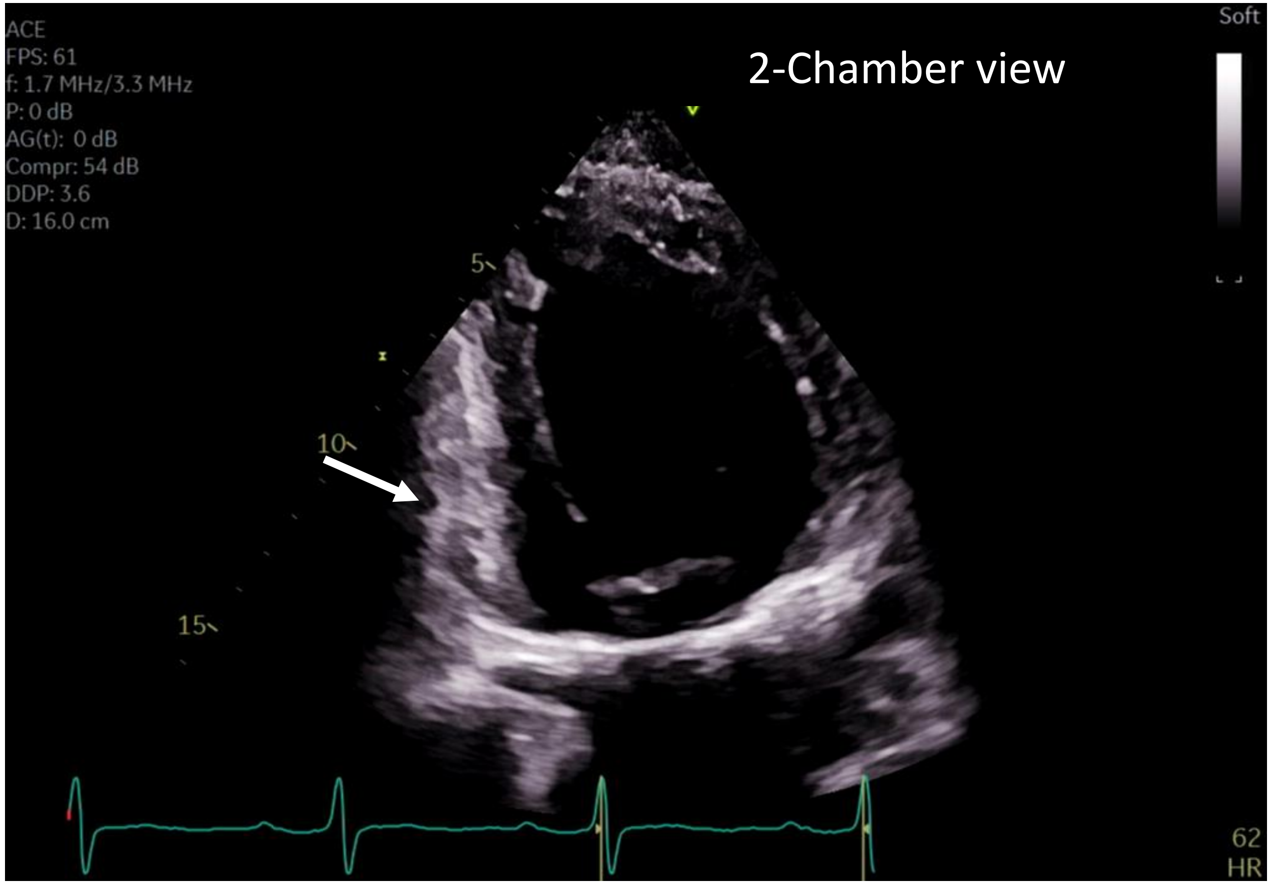

Wall Motion Abnormality
****Segment 4****

Case: Scar Map-Echo Wall Motion Abnormality

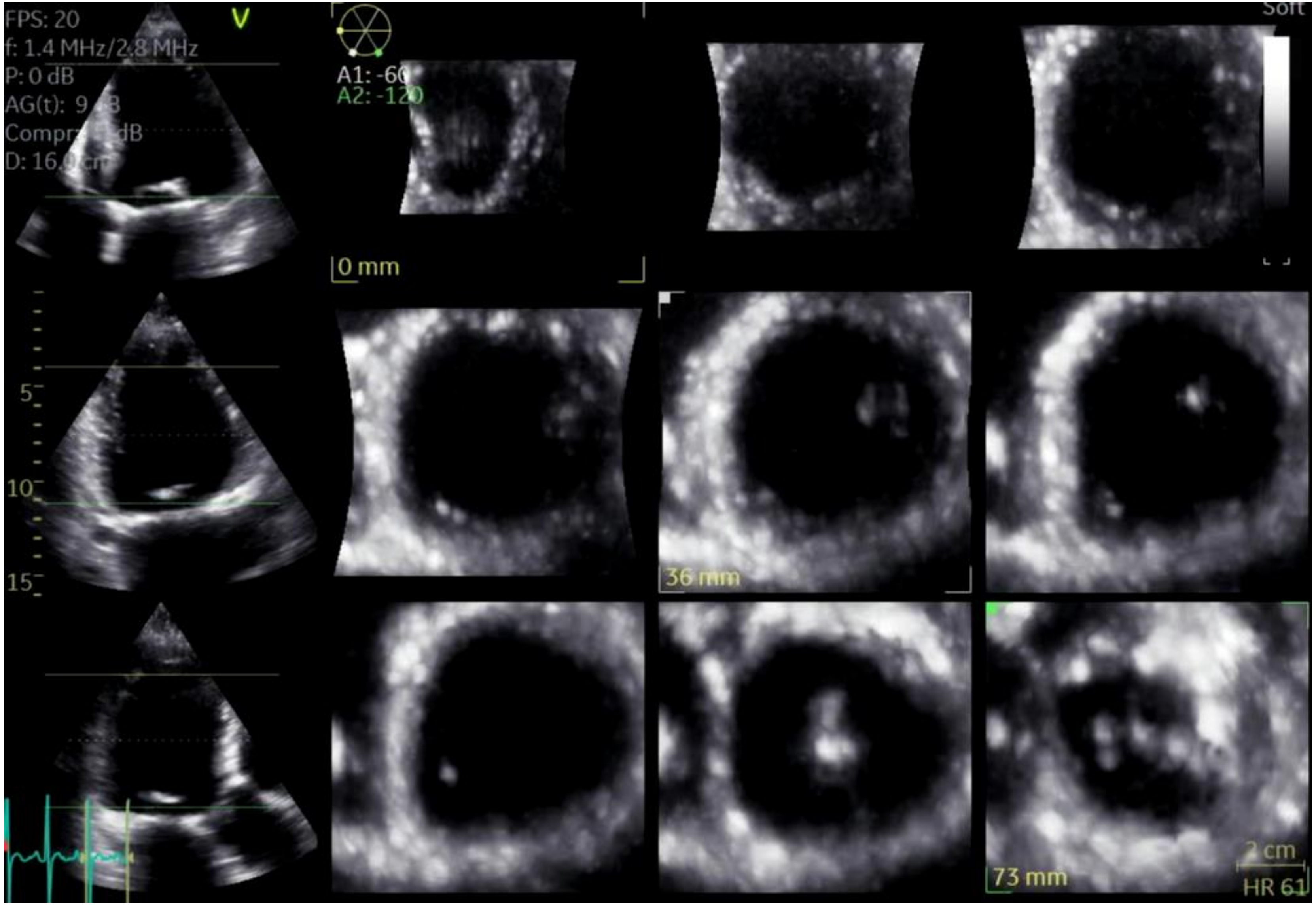

Apex
Normal

Mid
Segments 9,10

Base
Segments 4,5

<u>Case:</u> Scar Map-Echo Wall Motion Abnormality (strain)

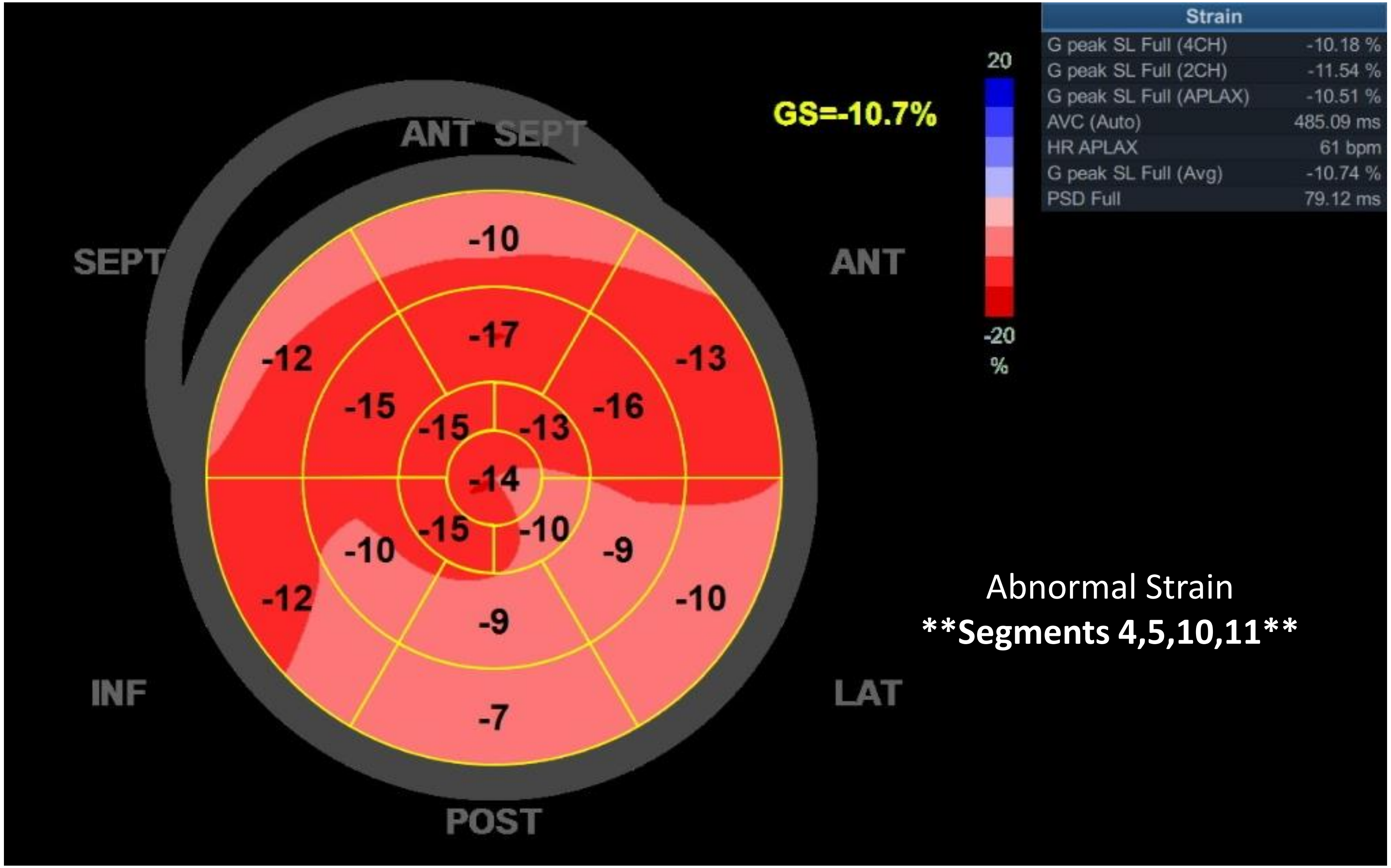

Abnormal Strain
Segments 4,5,10,11

Case 2

Abbreviated Clinical History

54 year old with NICM, NYHA class 2 HF, frequent ICD shocks

- Arrested at home in 2010 → CPR, followed by ICD
- 2013—Endo VT RFA (9 VTs induced, targeted LV+RV septum)
- 2014—Endo+Epi RFA (Epi normal→ targeted LV septum)
- 2016—Endo RFA (No VT, only VF→ targeted mid-apical LV septum)
- 2017—Seq/Sim Unipolar RF (targeted RV basal septum, LVOT)
- 2018—HD grid mapping (targeted basal-mid septum, 50W, 0.5ns, Bipolar)
- 2018—Alcohol septal ablation, 1st septal perforator

Abbreviated Medical History

- Antiarrhythmics:
  - Presently on Amio + Mex

- LVEF 20%

TARGETING DATA FORM

- Electrical Mapping
  - ☒ 12-lead Electrocardiogram (VT exit site)
  - ☒ Noninvasive Electrocardiographic Imaging (VT exit site)
  - ☒ Recent Invasive Catheter Map (activation, pace-map, prior radiofrequency ablation)

- Ventricular Scar Mapping
  - ☒ Echocardiogram (regional wall motion abnormality)
  - ☒ Nuclear Perfusion (non-viability)
  - ☒ PET Scan (inflammation)
  - ☒ Magnetic Resonance (gadolinium enhancement, wall motion abnormality, wall thinning)
  - ☒ Computed Tomography (wall thinning)
  - ☒ Recent Invasive Catheter Map (low amplitude electrograms)

Case: Electrical-12-lead Electrocardiogram (VT1 exit site)

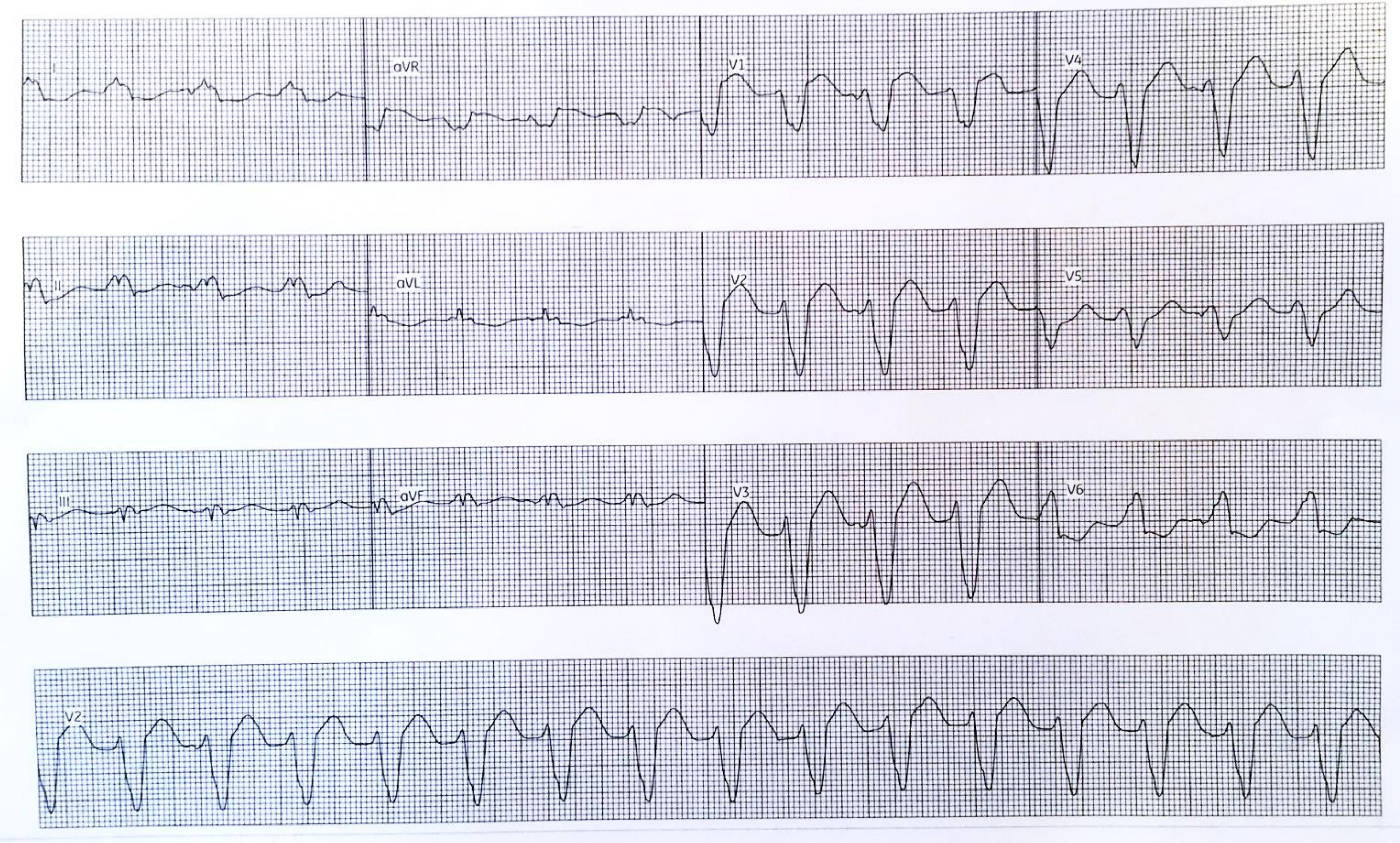

Case: Electrical-VT1 Catheter Activation Map

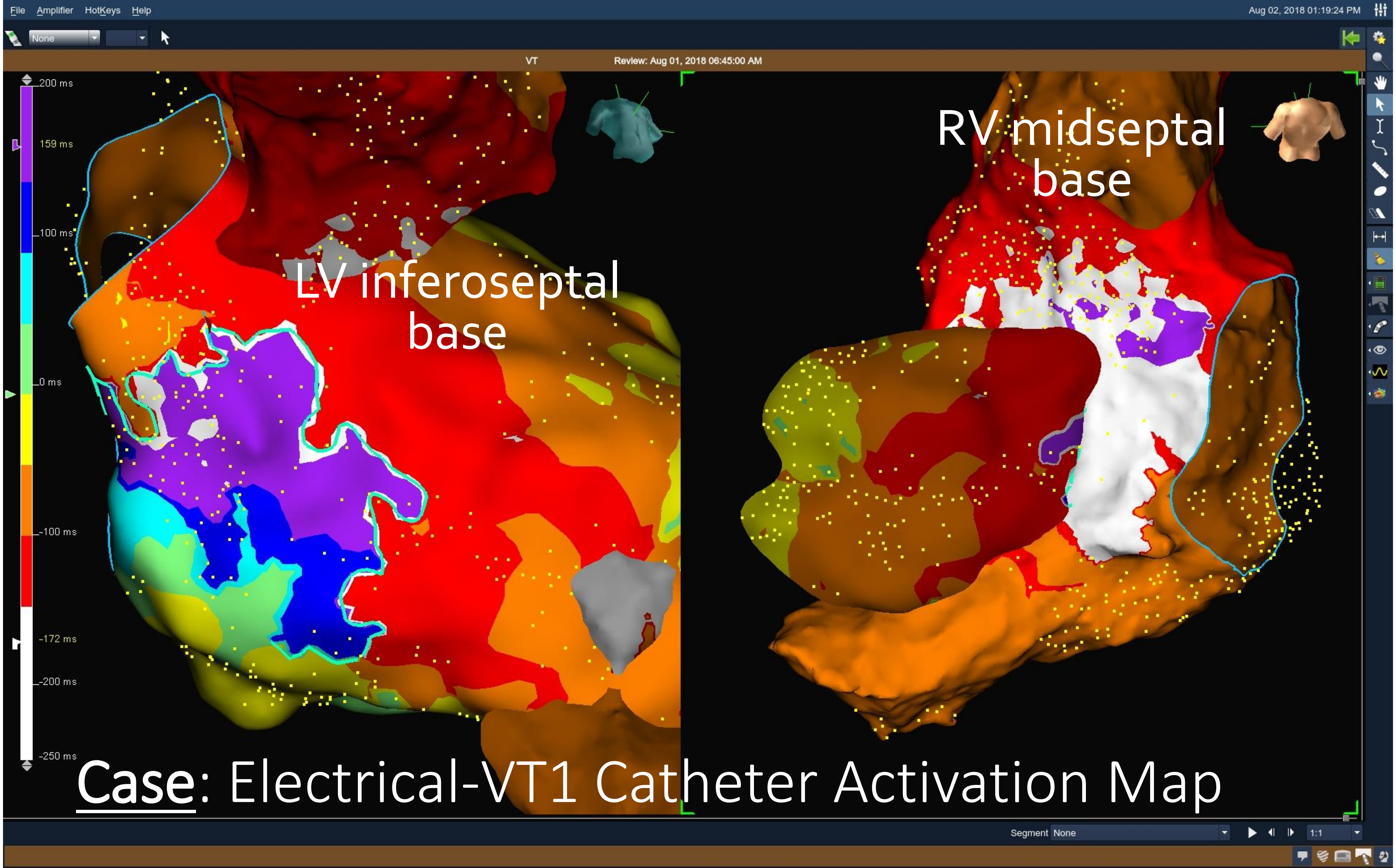

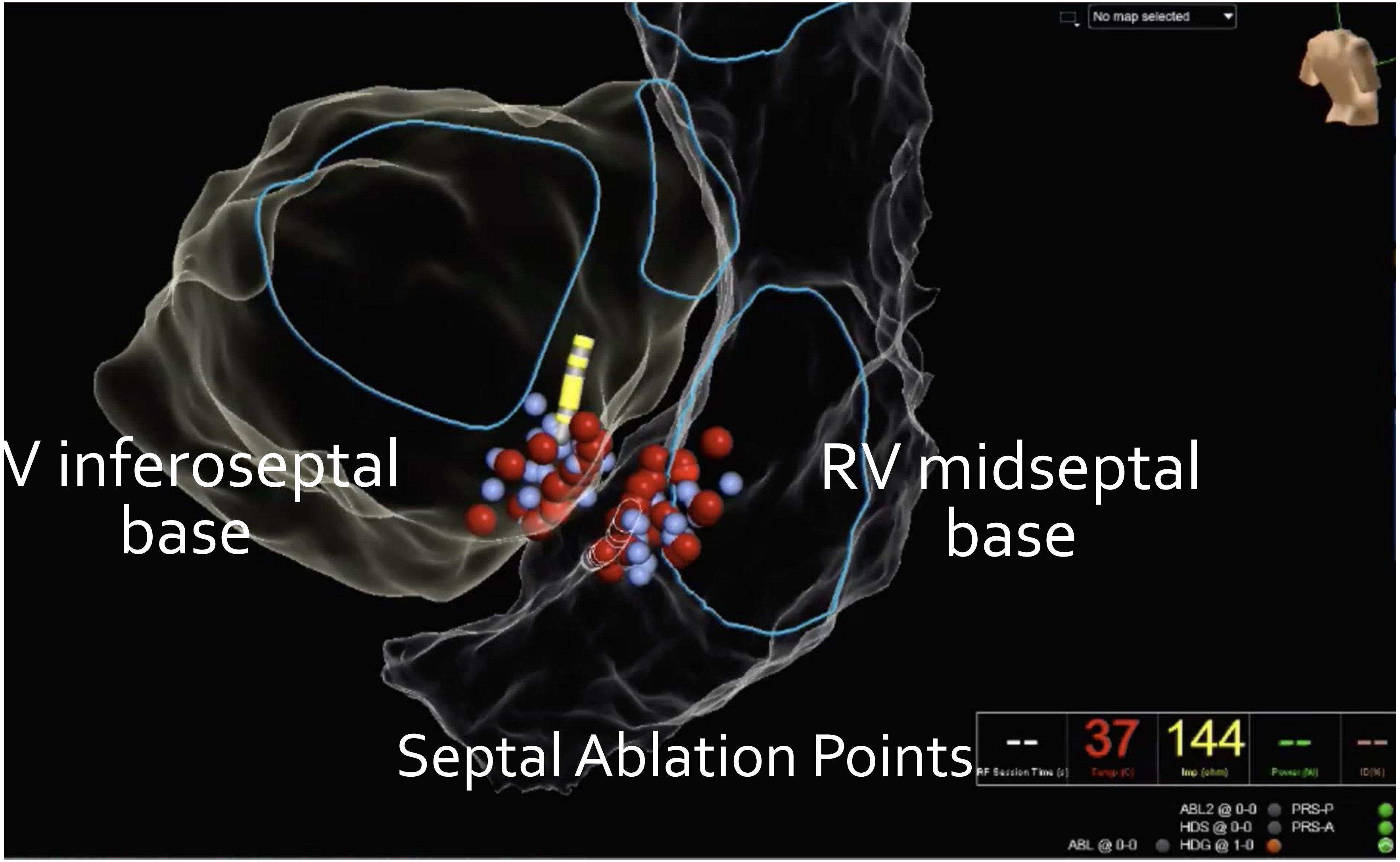

Case: Electrical-VT1 Catheter Ablation Points

<u>Case</u>: Electrical-VT1 Electrocardiographic Imaging

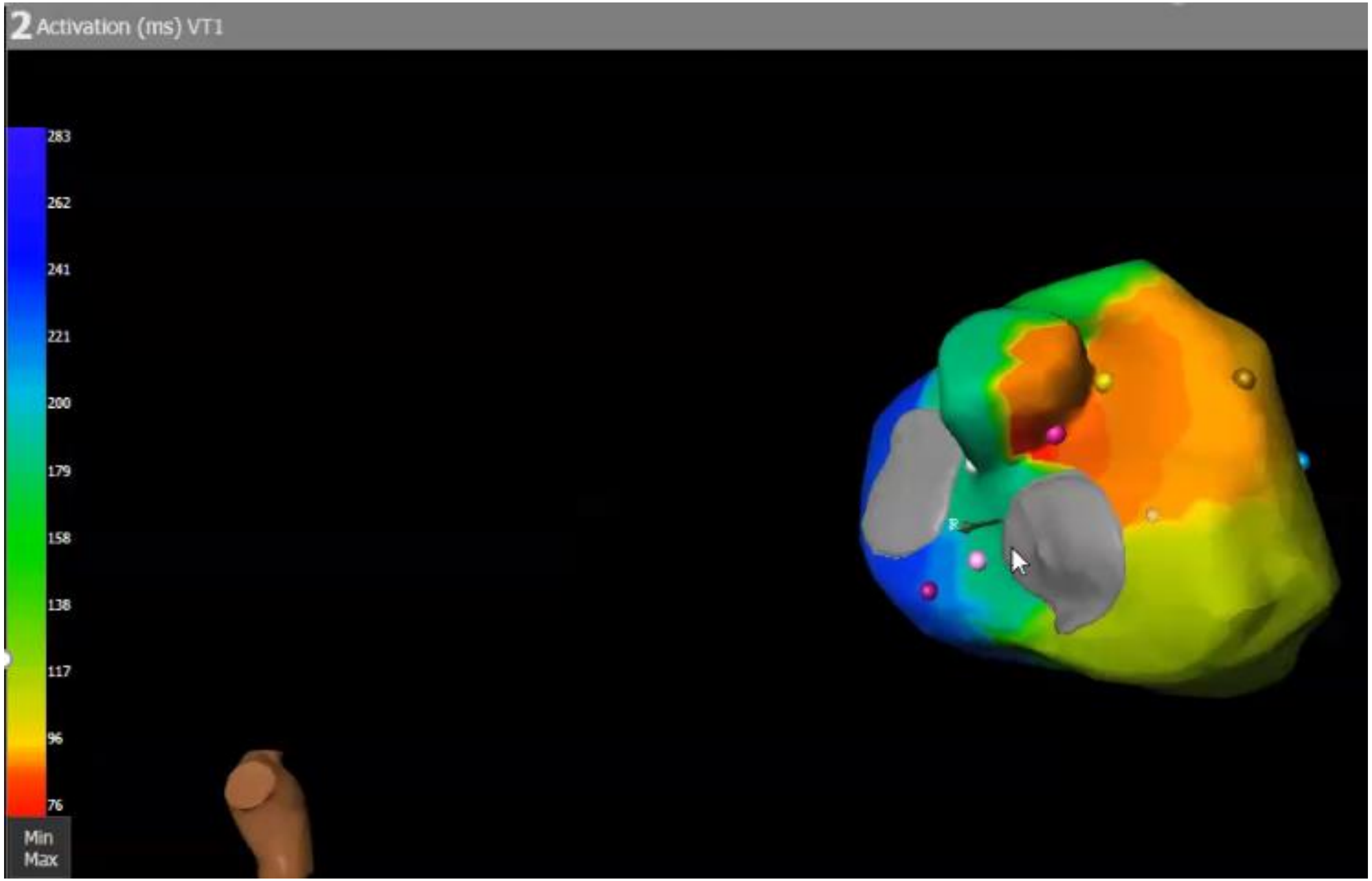

Case: Electrical-VT1 Electrocardiographic Imaging

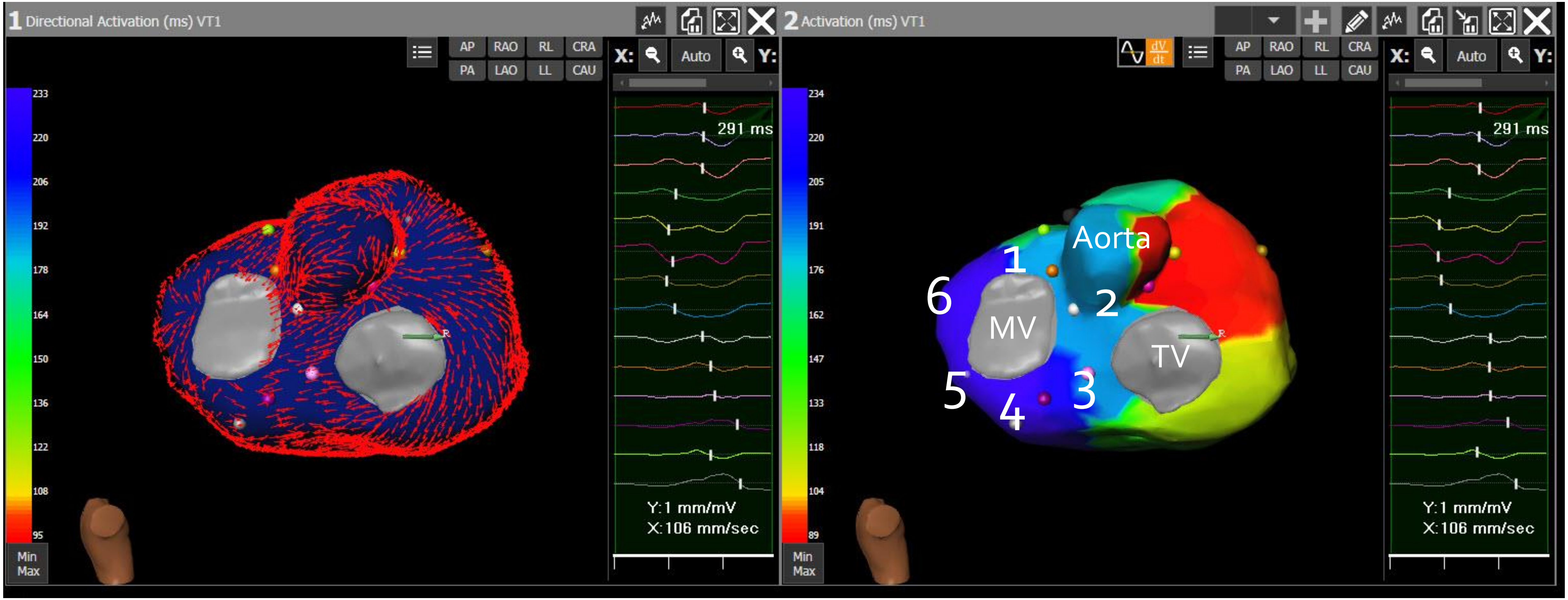

Case: Electrical-12-lead Electrocardiogram (VT2 exit site)

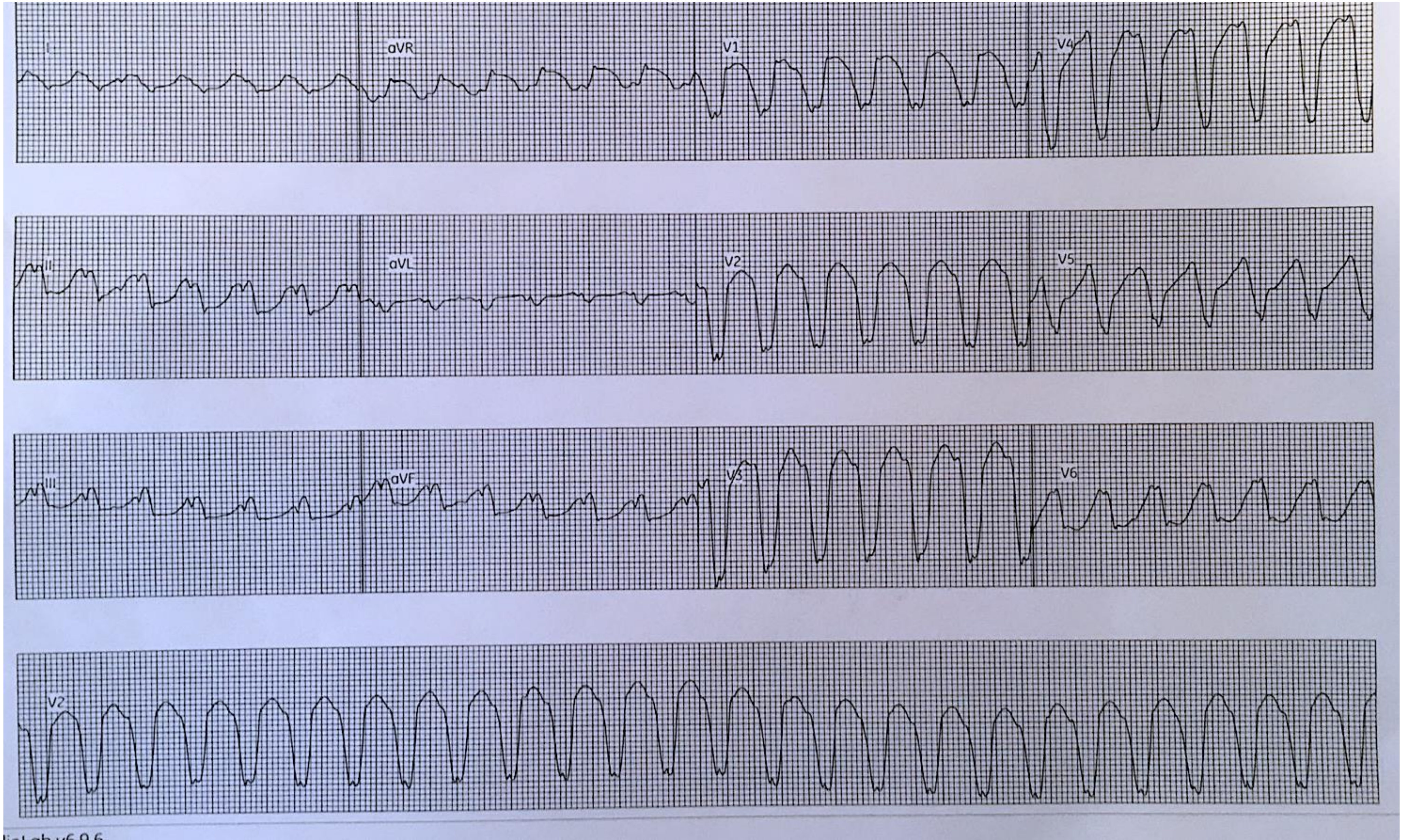

Case: Electrical-VT2 Electrocardiographic Imaging

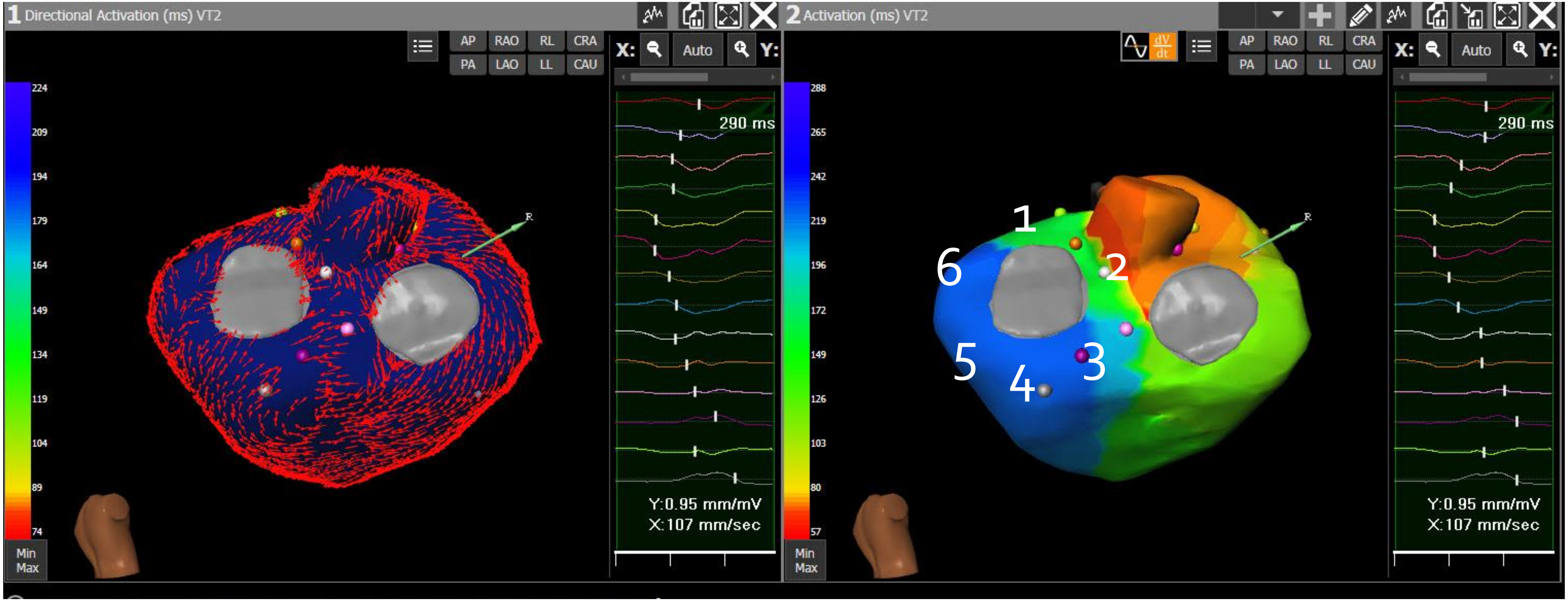

Case: Electrical-12-lead Electrocardiogram (VT3 exit site)

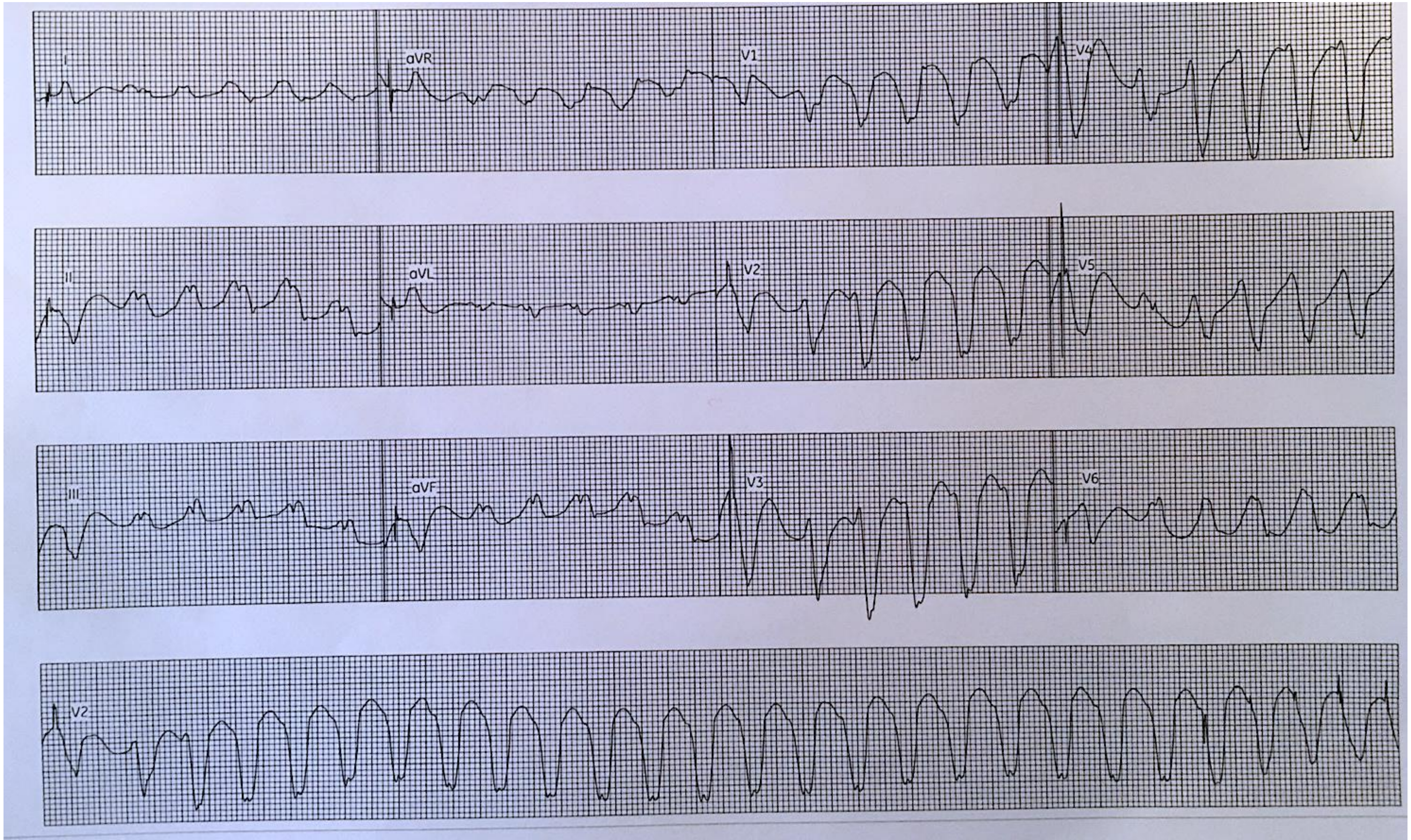

Case: Electrical-VT3 Electrocardiographic Imaging

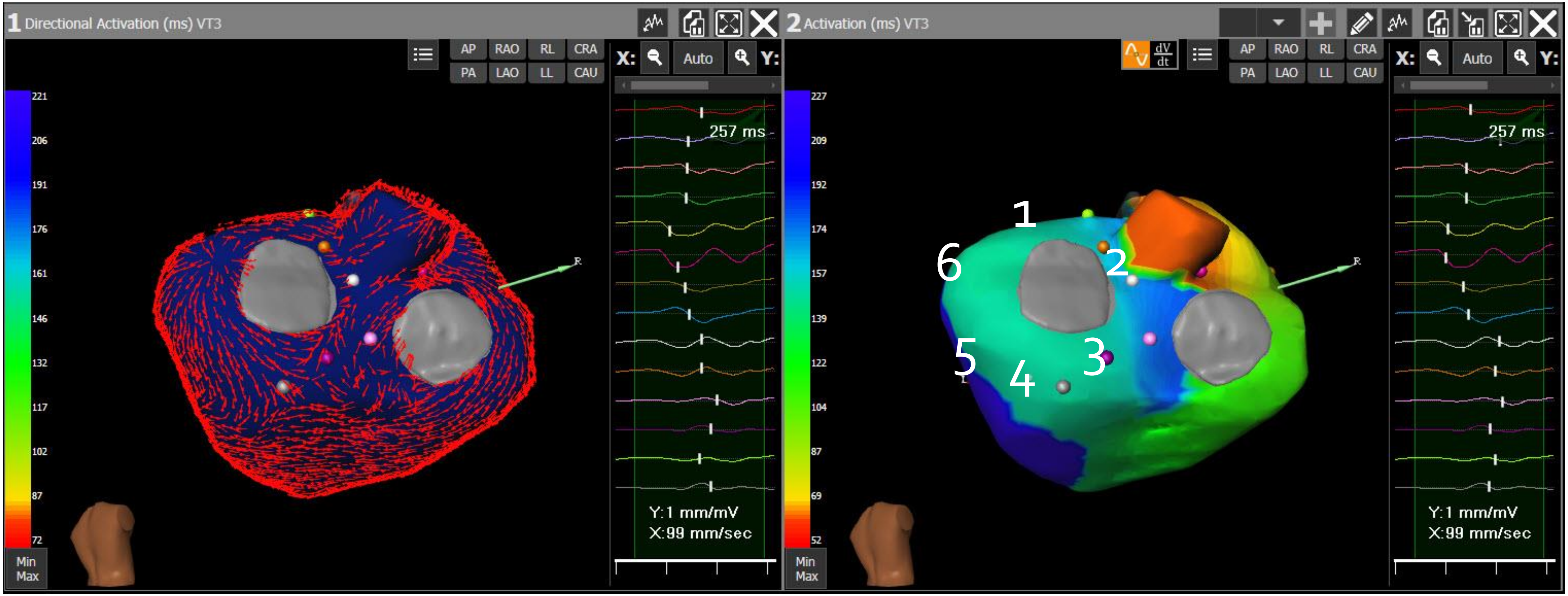

<u>Case</u>: Electrical-12-lead Electrocardiogram (VT4 exit site)

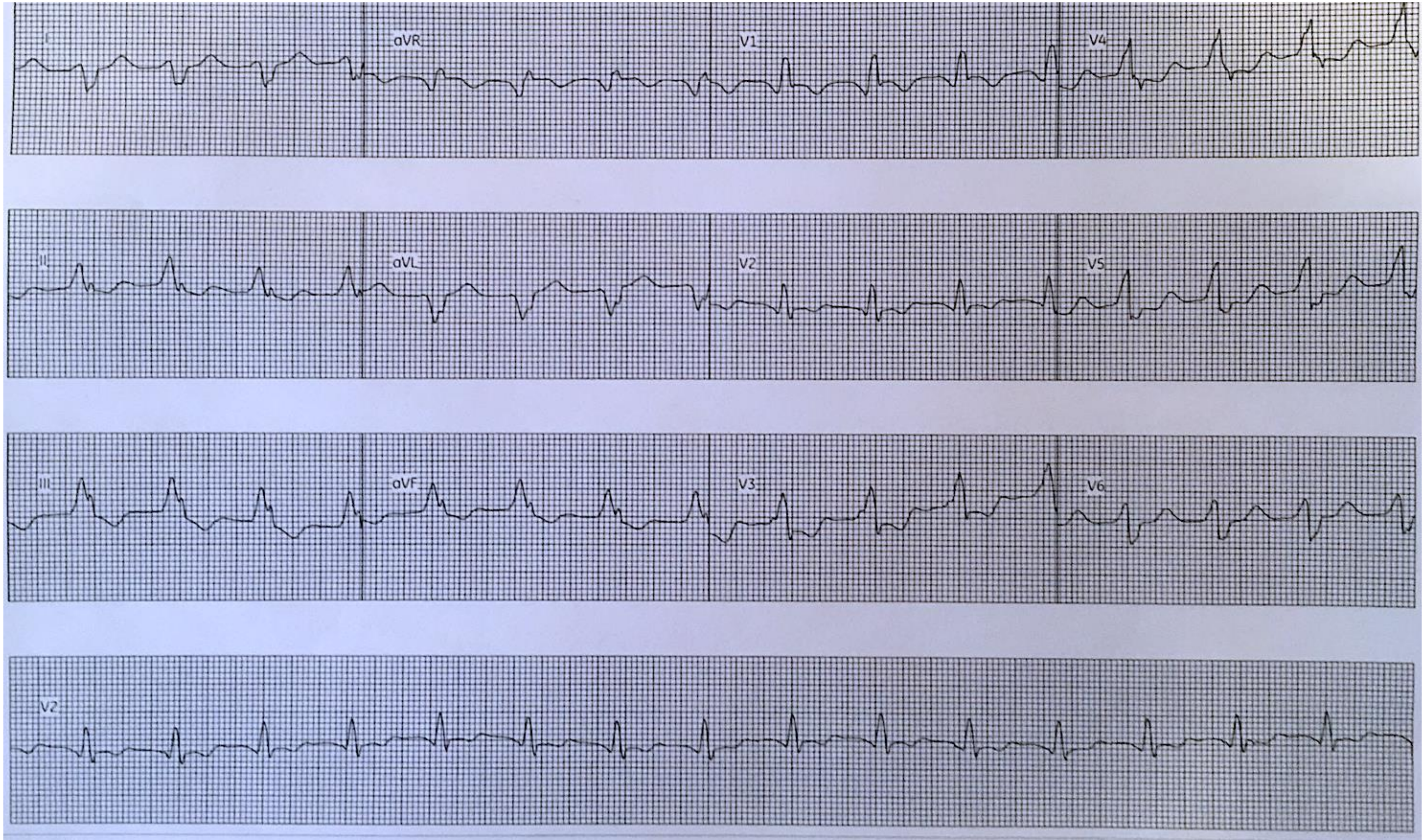

Case: Electrical-VT4 Electrocardiographic Imaging

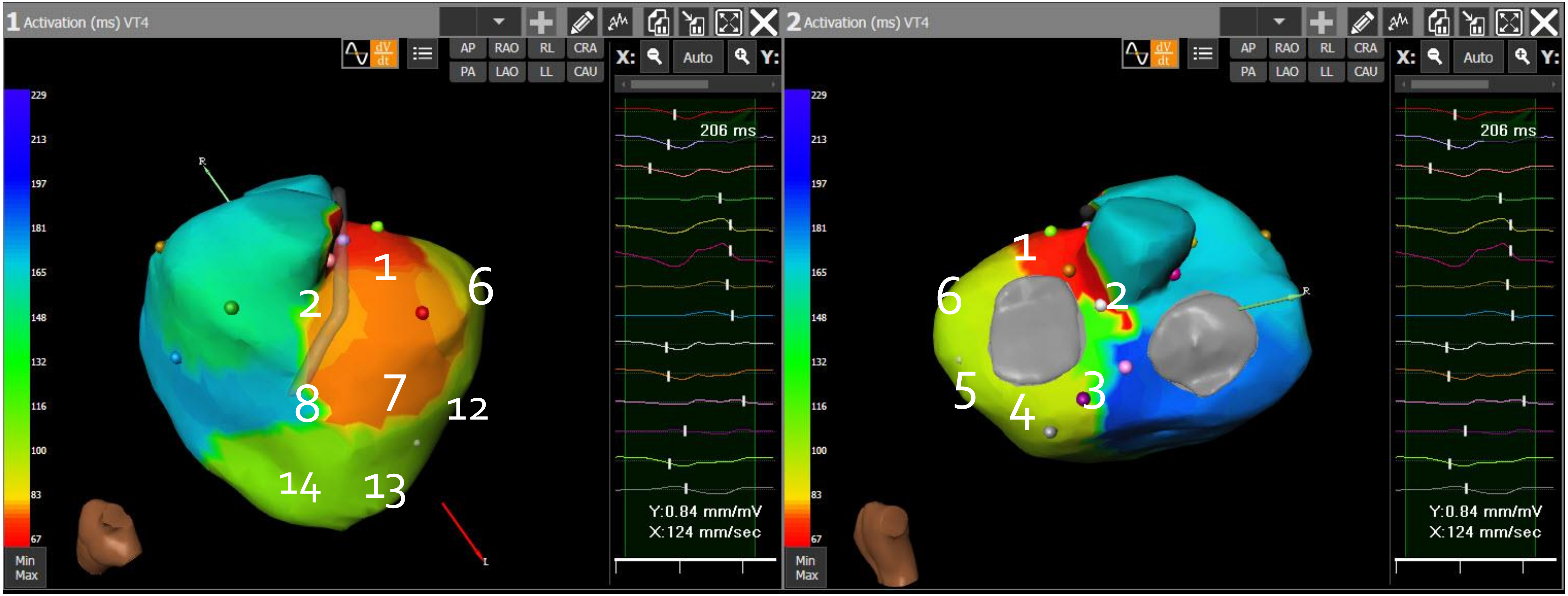

<u>Case</u>: Electrical-12-lead Electrocardiogram (VT5 exit site)

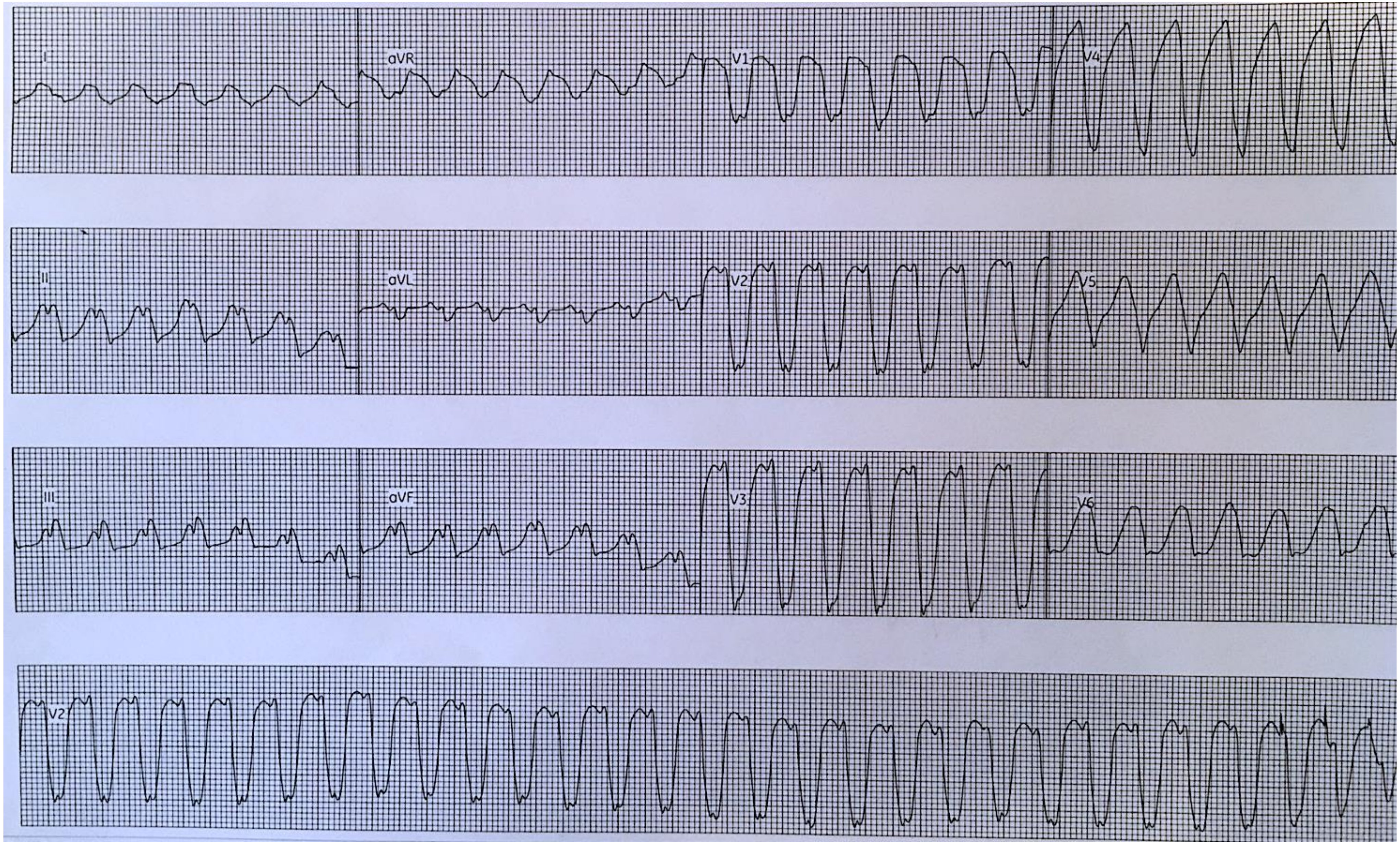

Case: Electrical-VT5 Electrocardiographic Imaging

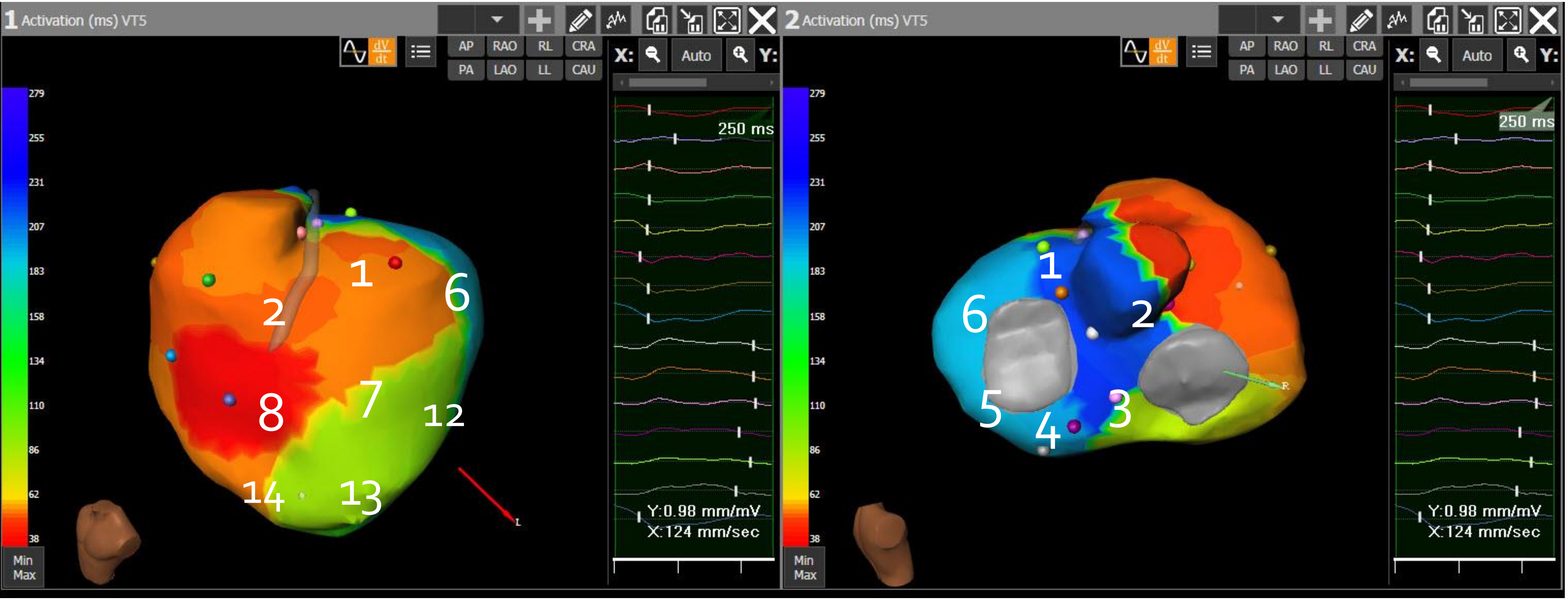

Case: Electrical-12-lead Electrocardiogram (VT6 exit site)

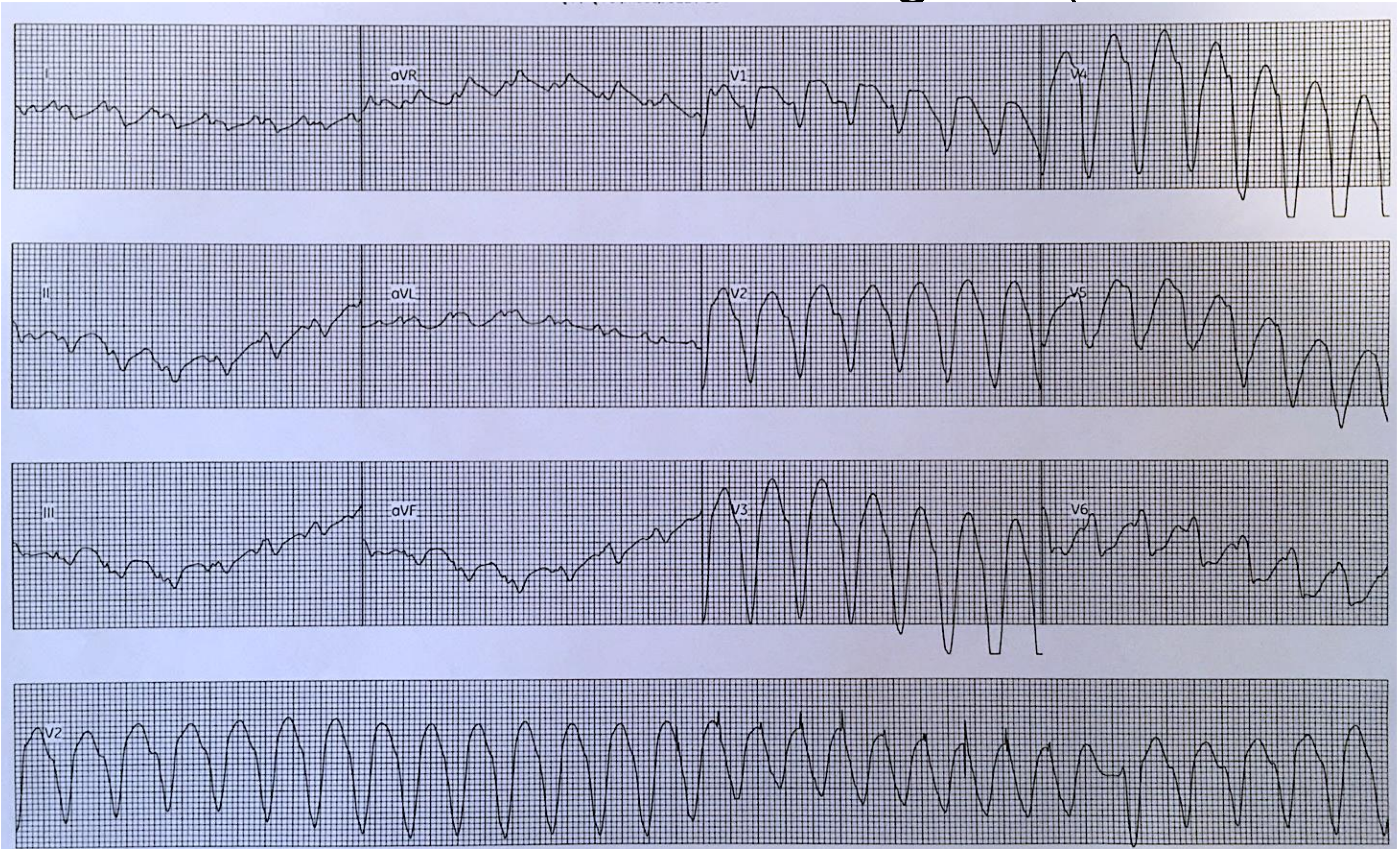

<u>Case</u>: Electrical-VT6 Electrocardiographic Imaging

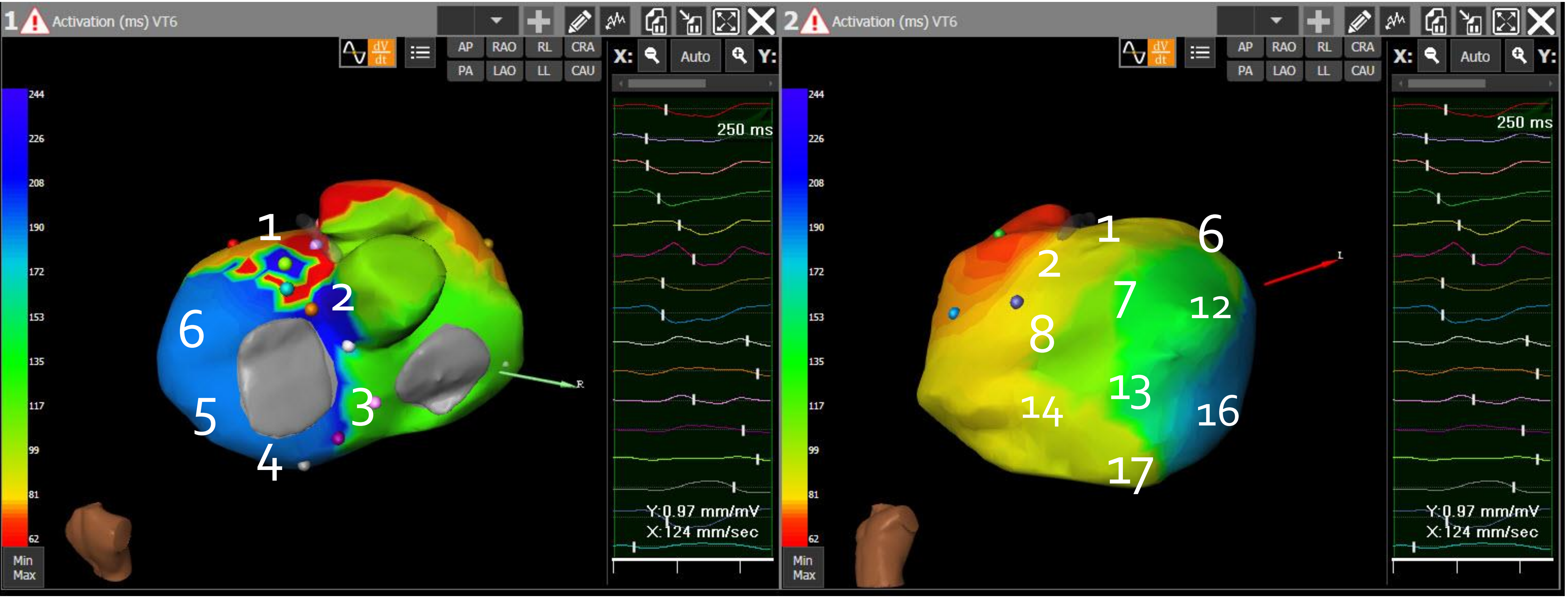

<u>Case</u>: Electrical-12-lead Electrocardiogram (VT7 exit site)

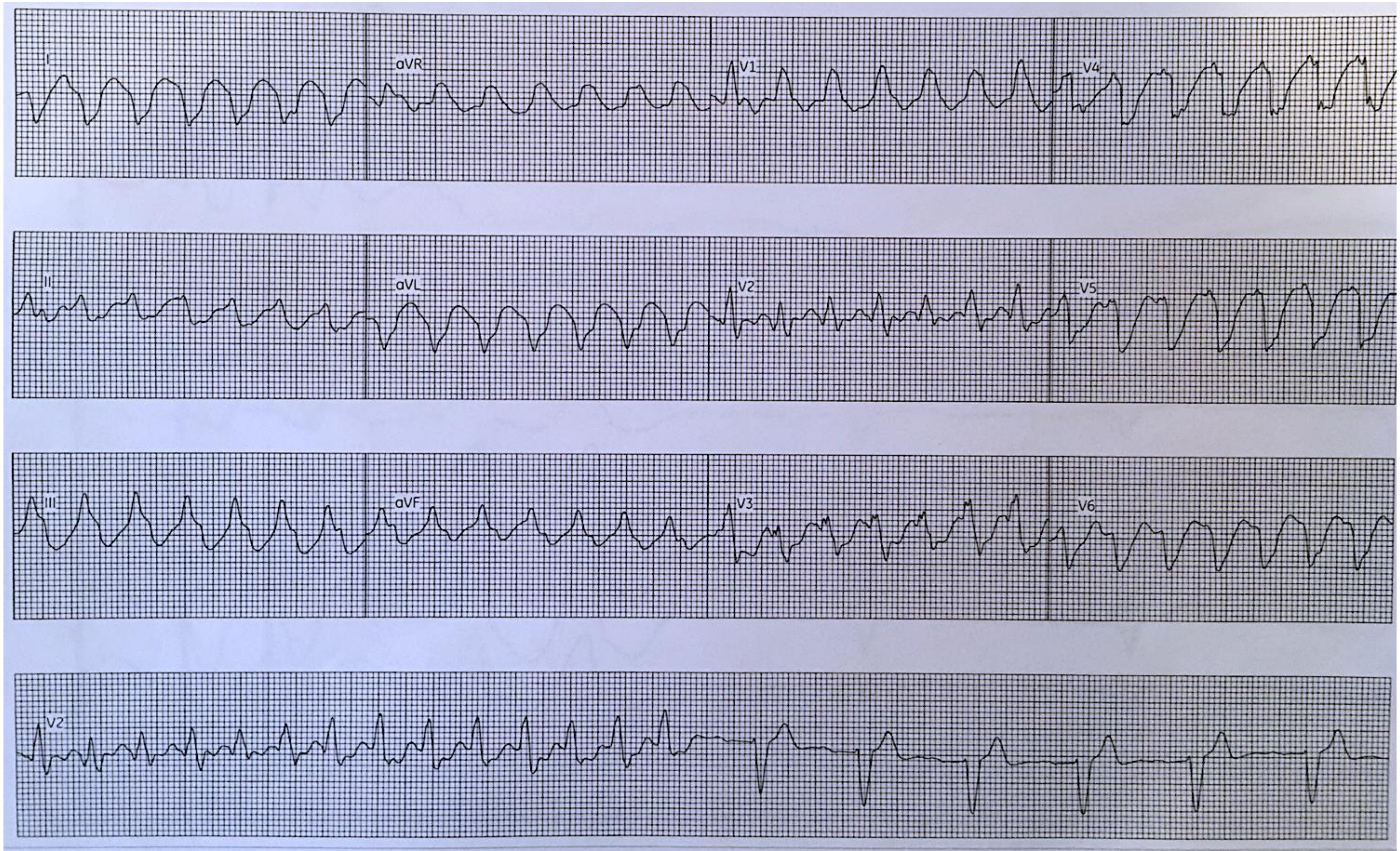

Case: Electrical-VT7 Electrocardiographic Imaging

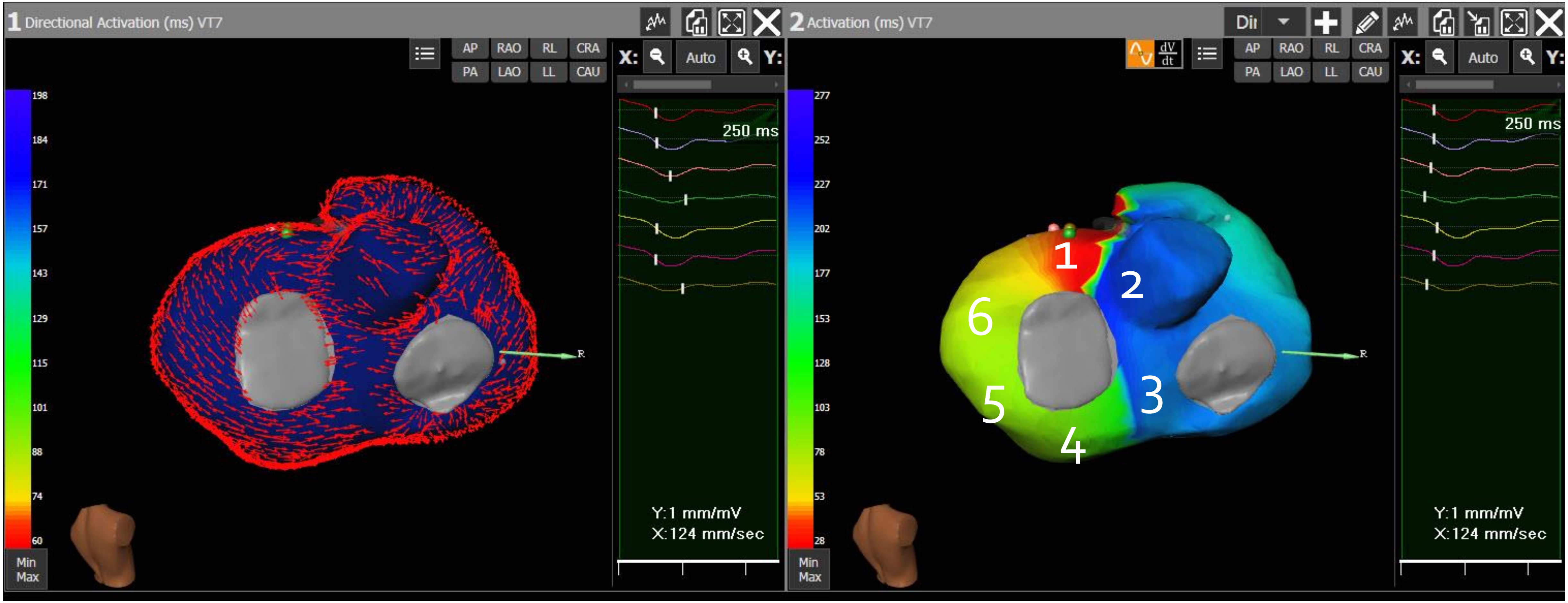

<u>Case:</u> Scar Map-Myocardial Resonance (MRI)

Basal segments: aorta, aortic valve, LV outflow, mitral valve

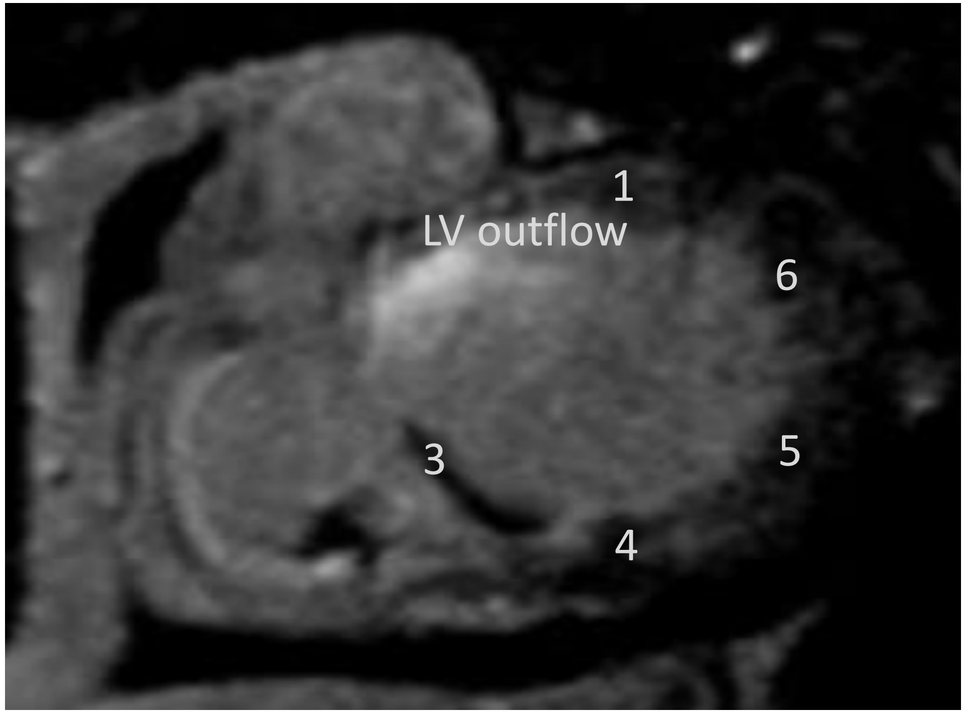

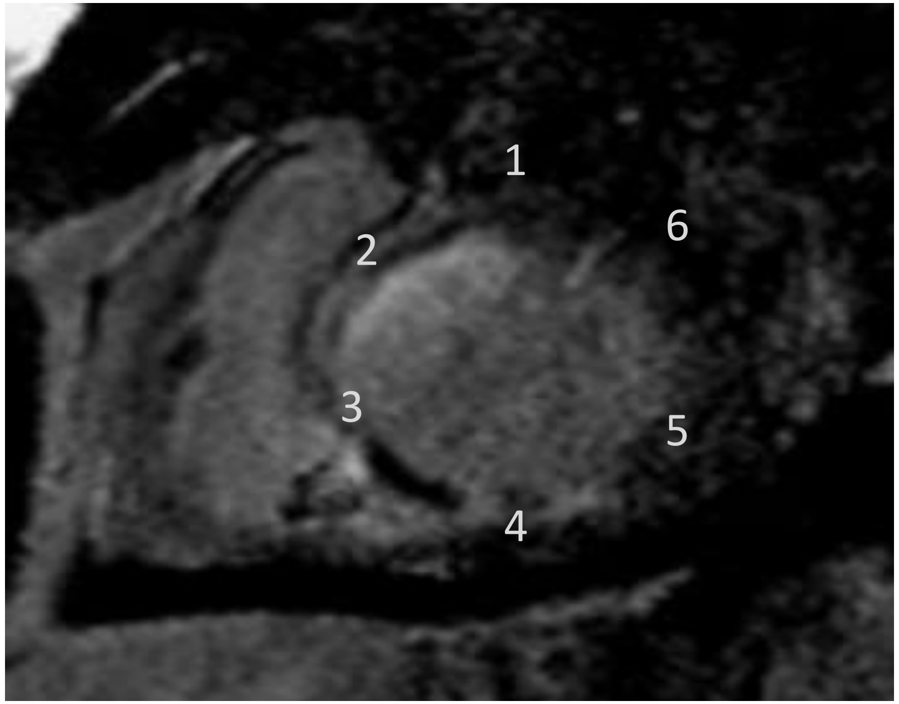

Gadolinium enhancement
Segments 2,3,4

<u>Case:</u> Scar Map-Myocardial Resonance (MRI)

Mid segments: papillary muscles

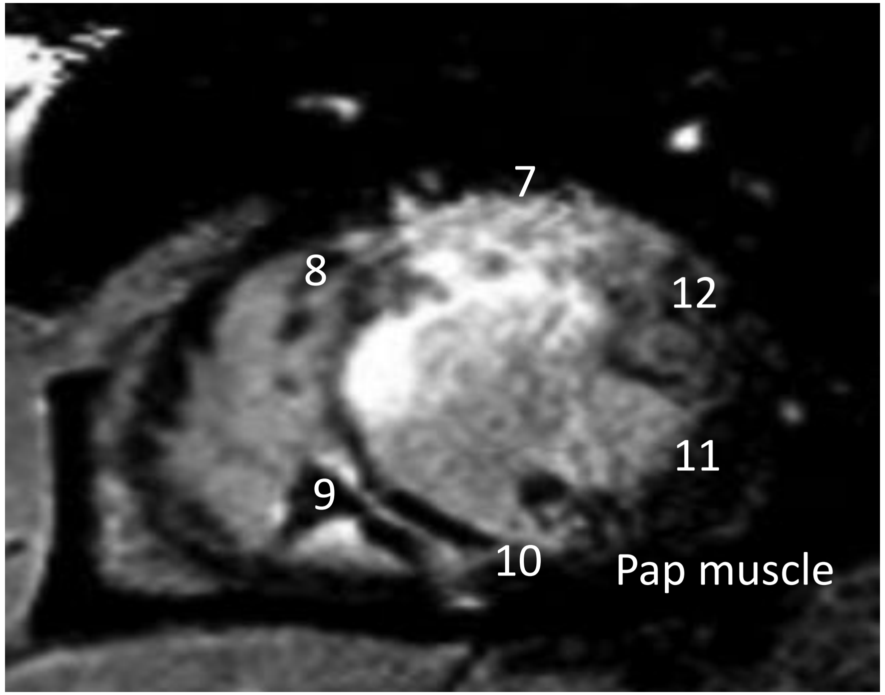

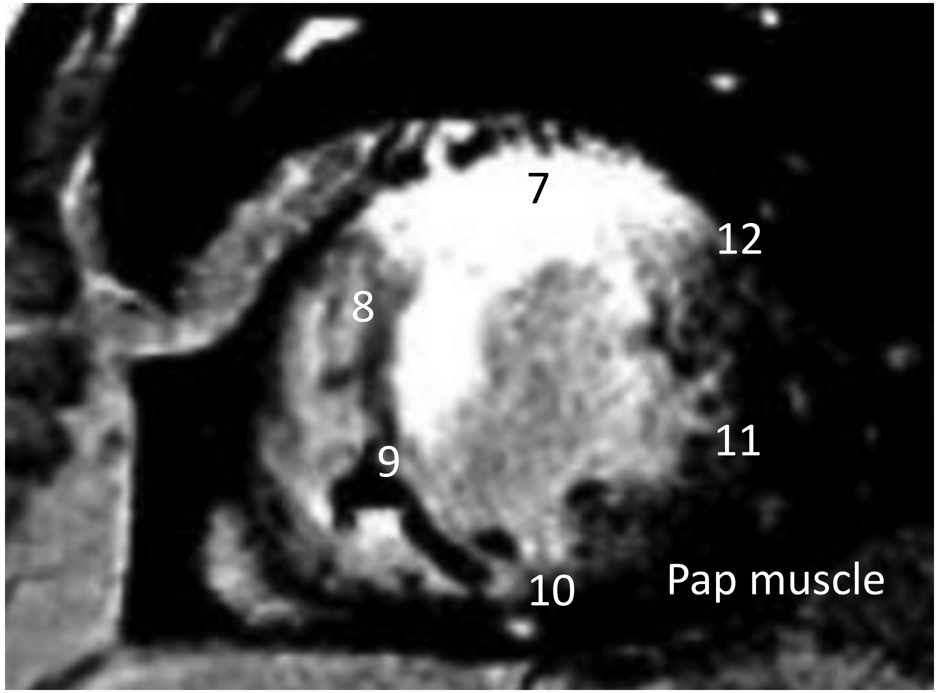

Gadolinium enhancement
****Segments 8,9****

Not interpretable:
Segment 7

Case: Scar Map-Myocardial Resonance (MRI)

Apical segments: no papillary muscle

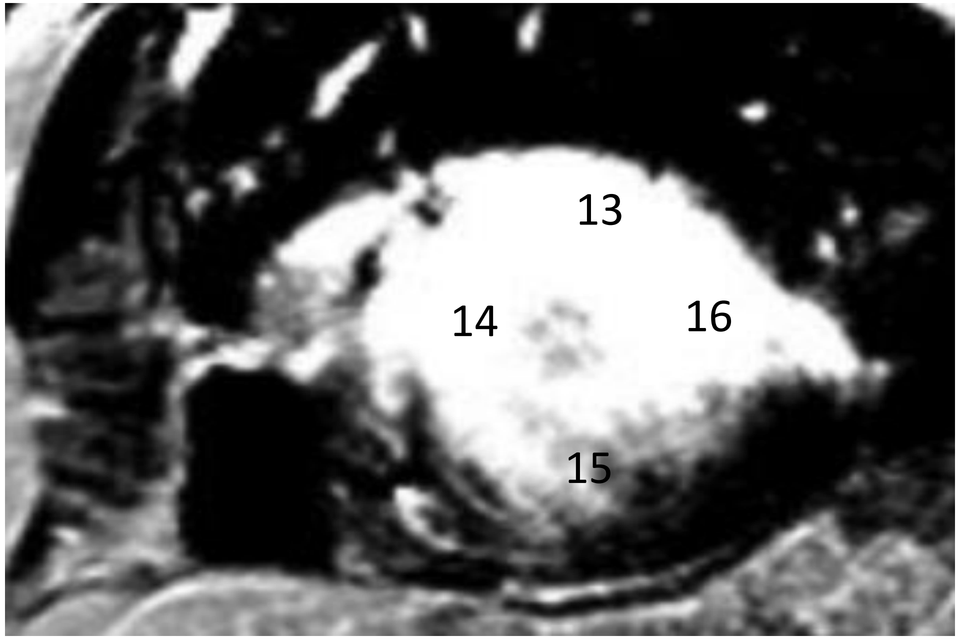

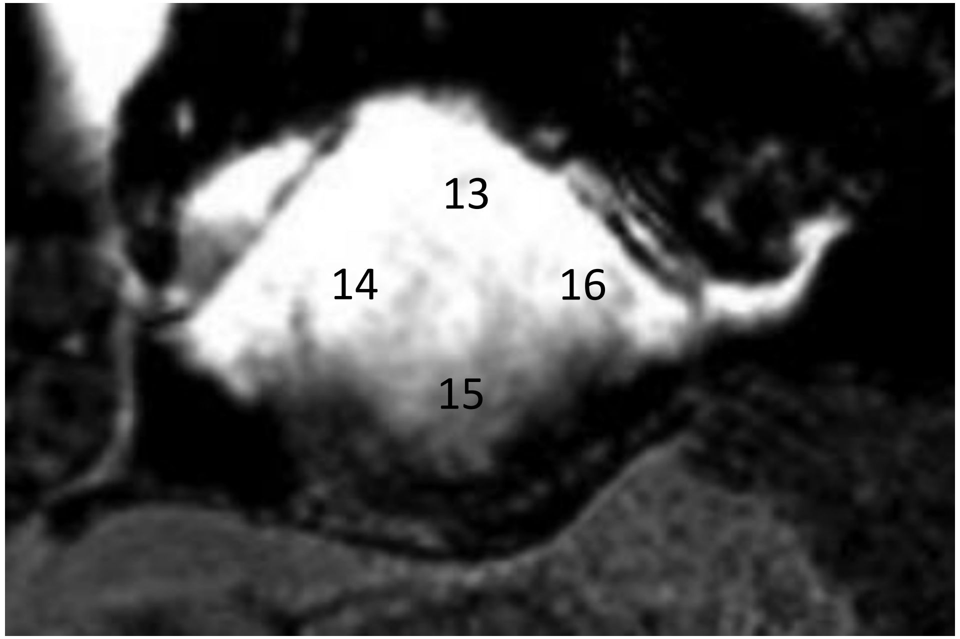

Gadolinium enhancement
None

Not interpretable:
Segment 13, 14, 16

<u>Case:</u> Scar Map-Myocardial Resonance (MRI)

Basal segments: aorta, aortic valve, LV outflow, mitral valve

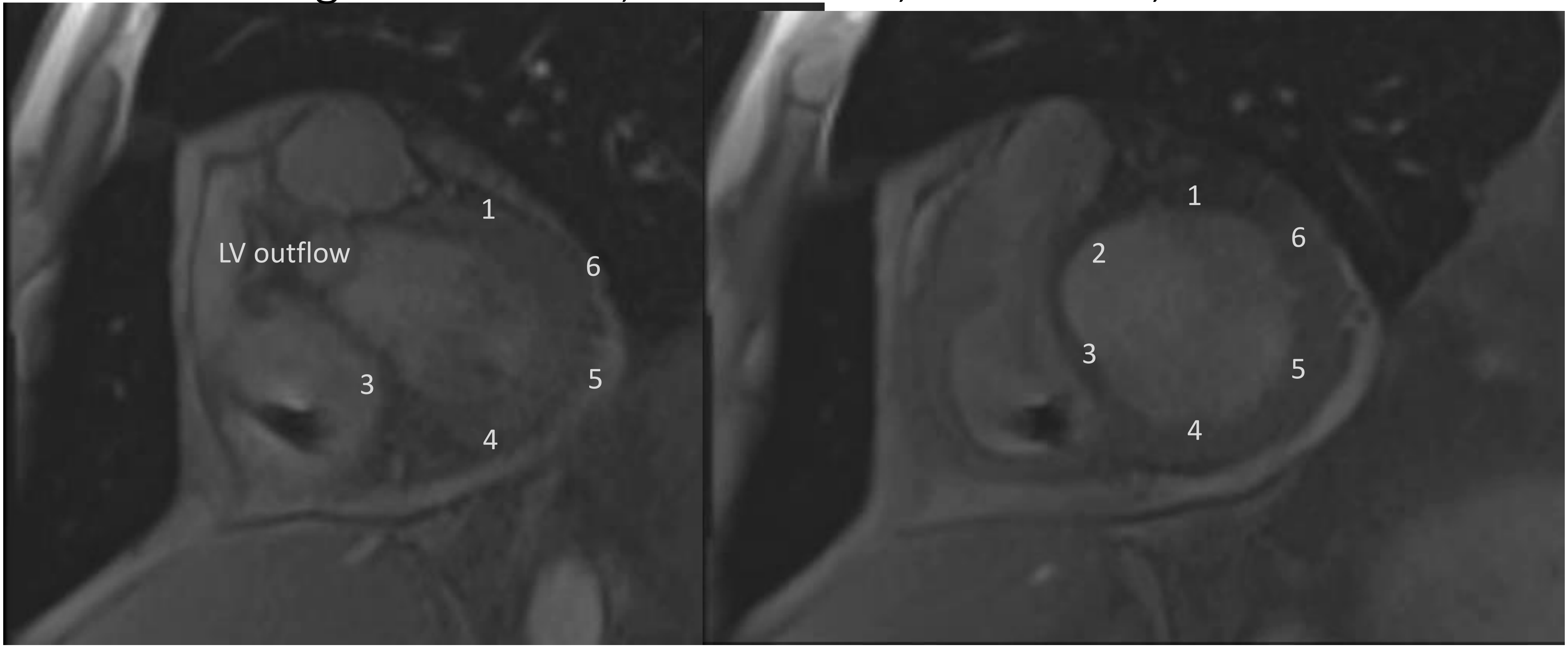

Wall motion abnormalities
Segments 2,3

Case: Scar Map-Myocardial Resonance (MRI)

Mid segments: papillary muscles

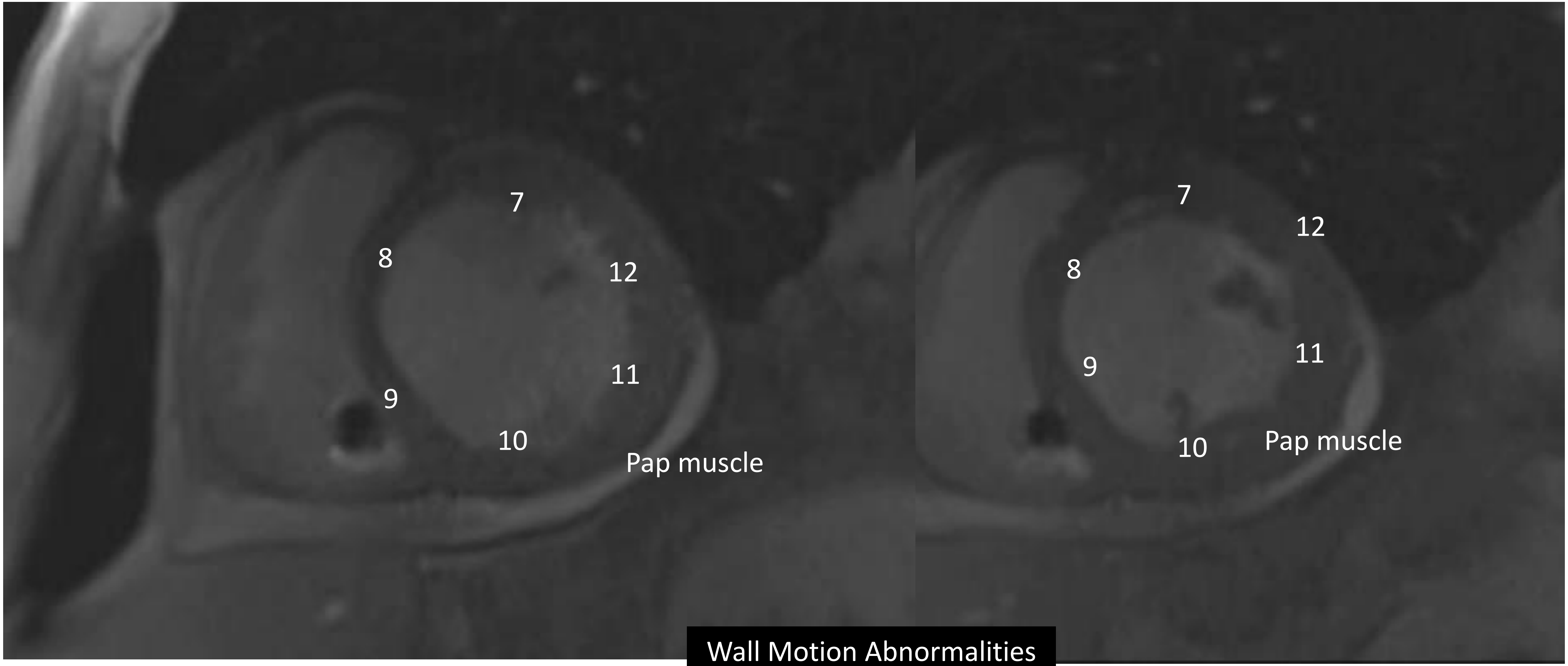

Wall Motion Abnormalities
****Segments 8,9****

Case: Scar Map – PET Inflammation

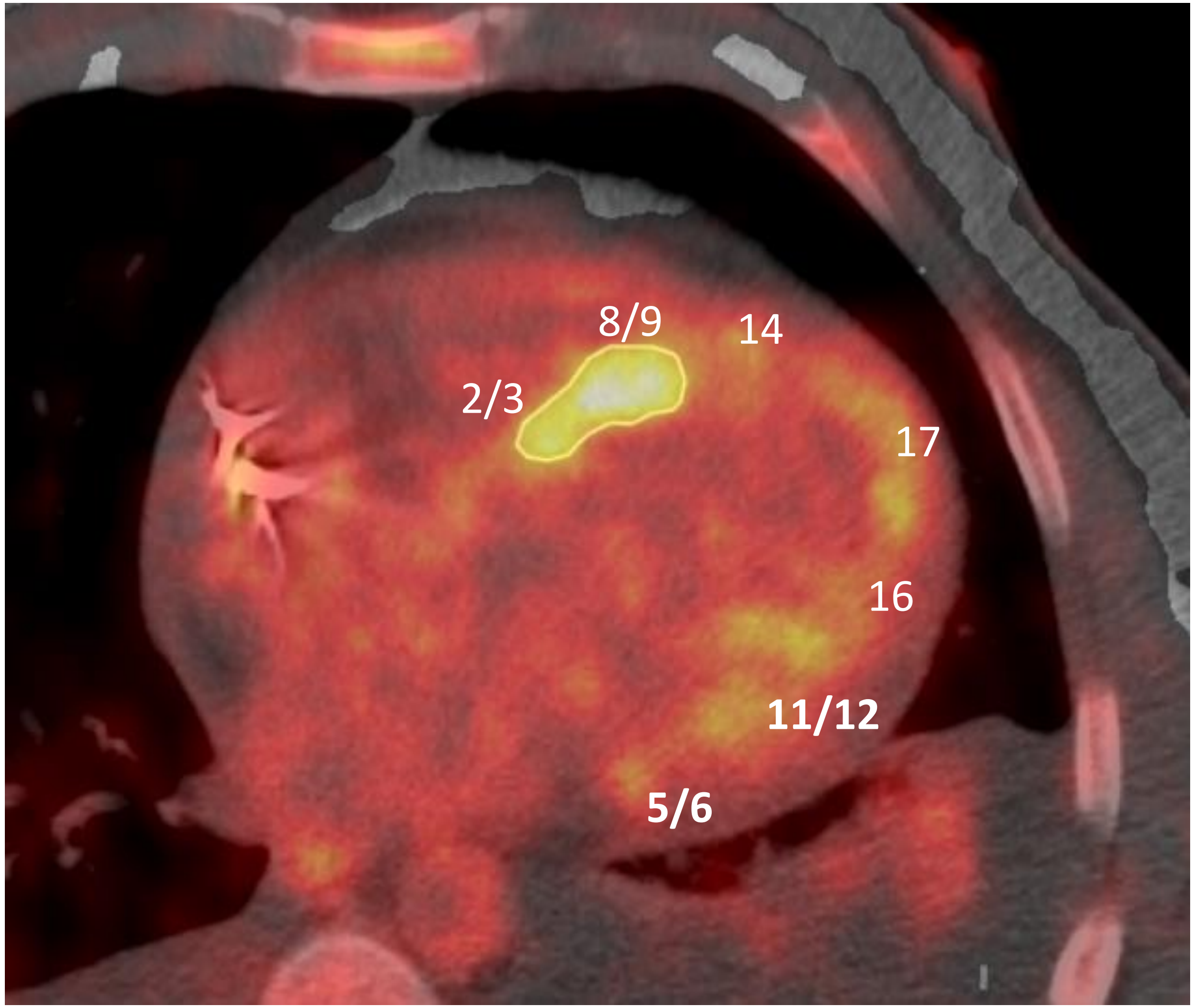

Inflammation
****Segments 2,3,8,9****

Case: Scar Map – SPECT Perfusion

Images not available
Decreased Perfusion
****Segments 2,3,8,9****

Case: Scar Map – Echocardiogram

Images not available
Wall Motion Abnormalities
****Segments 3,4,8,9,10,14****

<u>Case:</u> Scar Map – CT Scan

Images not available
Wall Abnormalities
****None****

Case 3

Abbreviated Clinical History

70 year old with NICM, NYHA class 2 HF, frequent ICD shocks

- 2002—Endo ablation, stayed on amiodarone
- 2009—Endo/Epi ablation, stayed on amiodarone
- 1/2019—Endo ablation: Lateral LV base. 2 other nonclinical VTs at end.
- 1/2019—Epi RFA ablation: Lateral LV mid & base
- 5/2019—Endo/Epi ablation: Large lateral scar. Multiple VTs, perimitral & peri-aortic. Procedure complicated by RV puncture, surgical repair via sternotomy.
- Currently on amiodarone and mexiletine
- LVEF 32%, moderate MR. LVEDD 6.5cm, LVESD 5.5cm.

TARGETING DATA FORM

- Electrical Mapping
  - ☒ 12-lead Electrocardiogram (VT exit site)
  - ☒ Noninvasive Electrocardiographic Imaging (VT exit site)
  - ☐ Recent Invasive Catheter Map (activation, pace-map, prior radiofrequency ablation)

- Ventricular Scar Mapping
  - ☒ Echocardiogram (regional wall motion abnormality)
  - ☒ Nuclear Perfusion (non-viability)
  - ☒ PET Scan (inflammation)
  - ☒ Magnetic Resonance (gadolinium enhancement, wall motion abnormality, wall thinning)
  - ☐ Computed Tomography (wall thinning)
  - ☐ Recent Invasive Catheter Map (low amplitude electrograms)

<u>Case</u>: Electrical-12-lead Electrocardiogram (VT1 exit site)

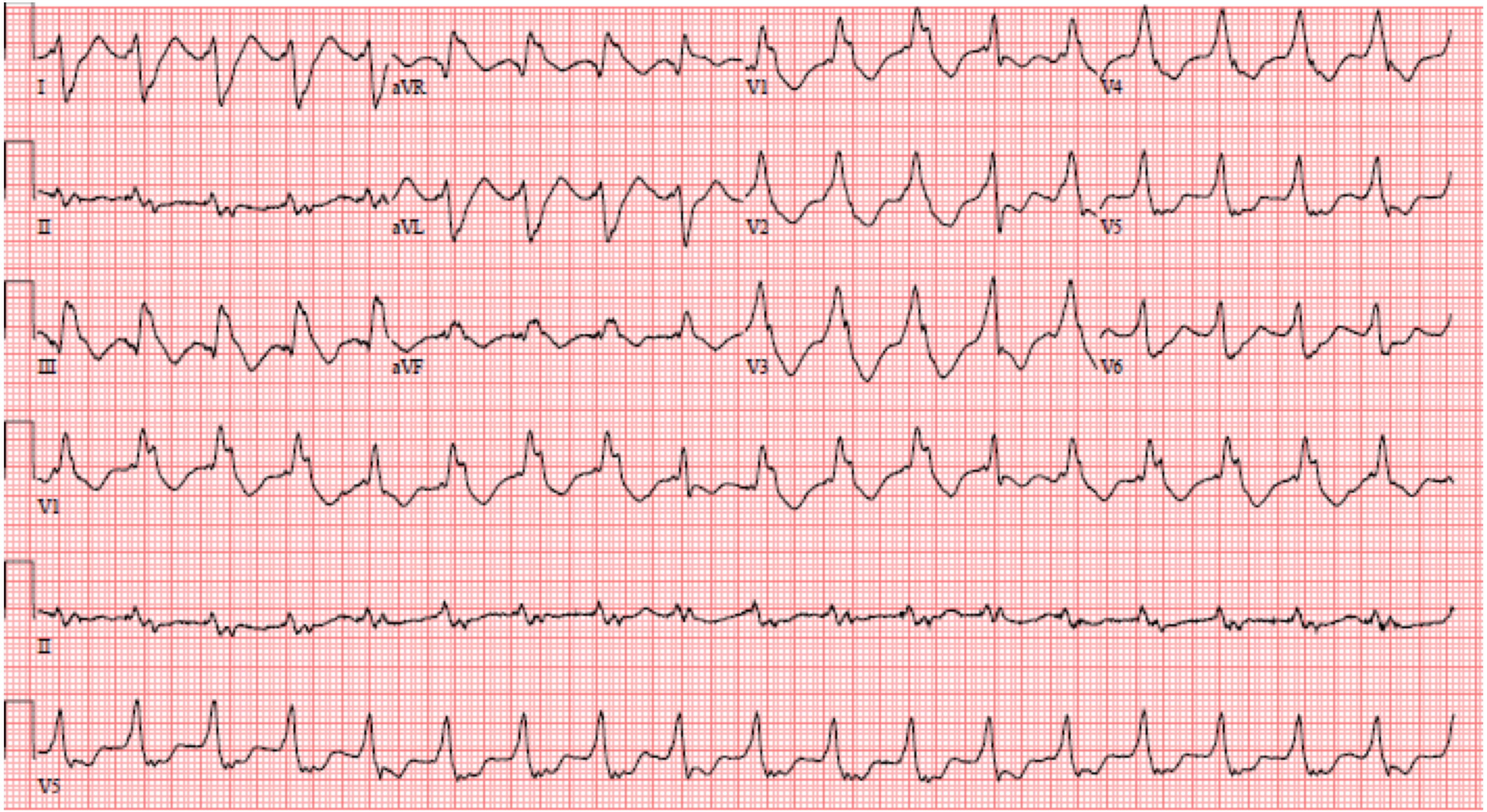

Case: Electrical-VT1 Electrocardiographic Imaging (Activation map, early = red)

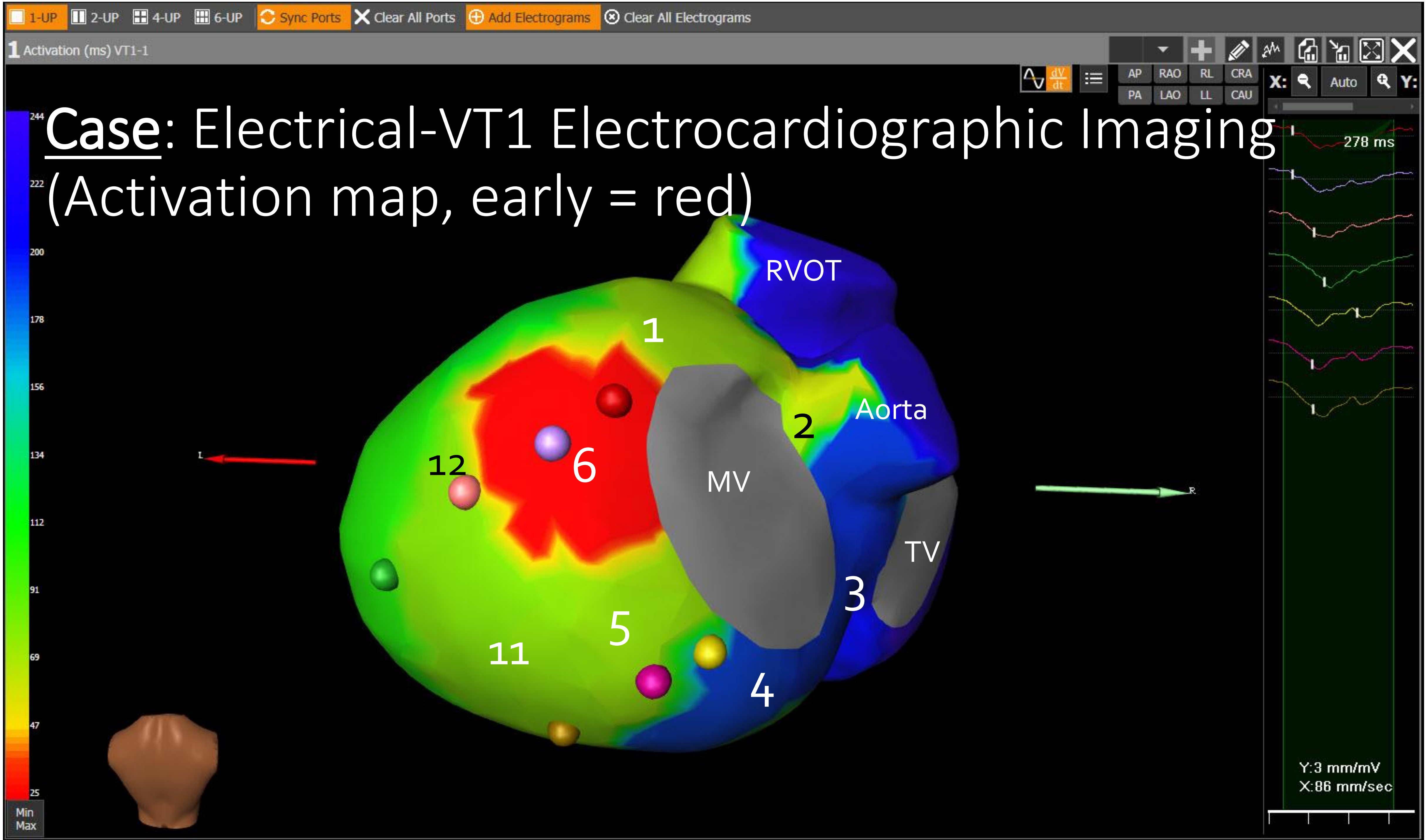

Case: Electrical-12-lead Electrocardiogram (VT4 exit site)

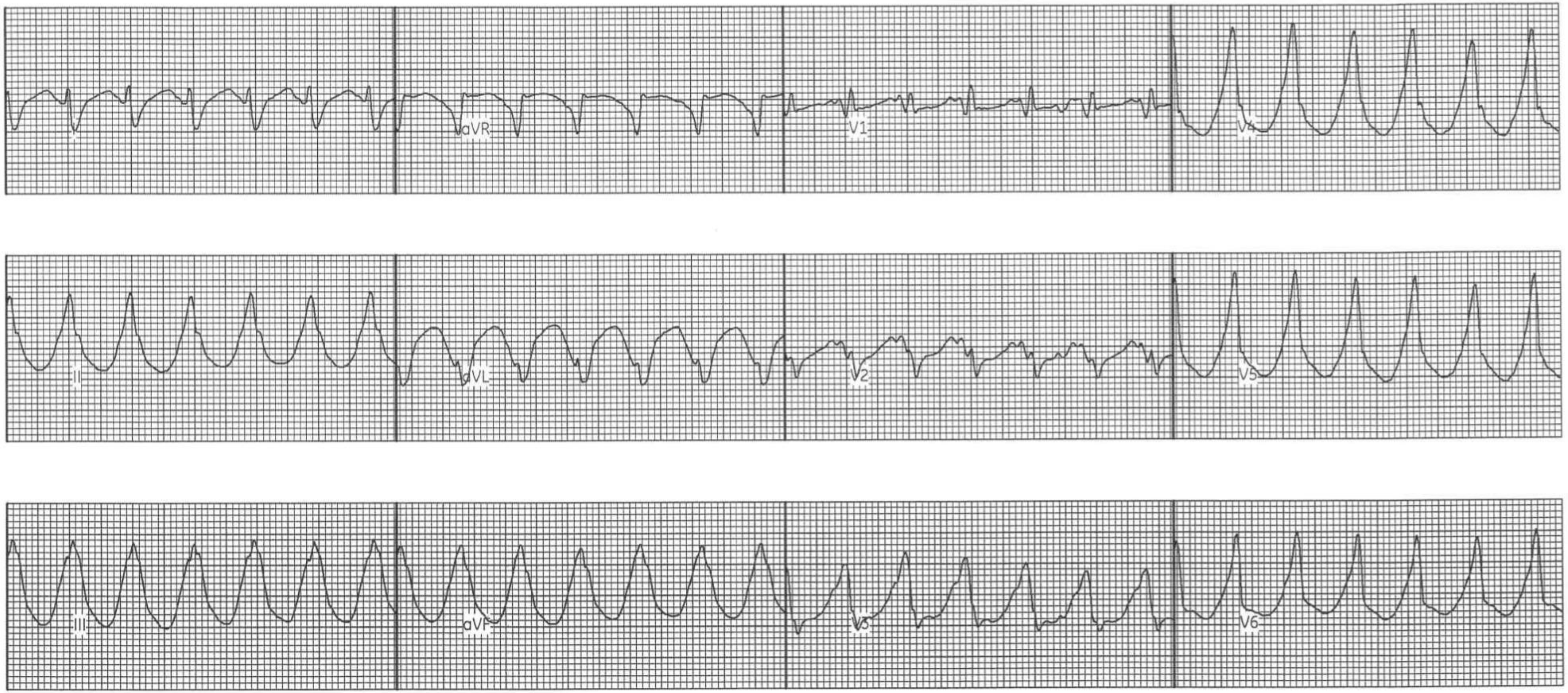

Case: Electrical-VT4 Electrocardiographic Imaging
Potential map, early = white

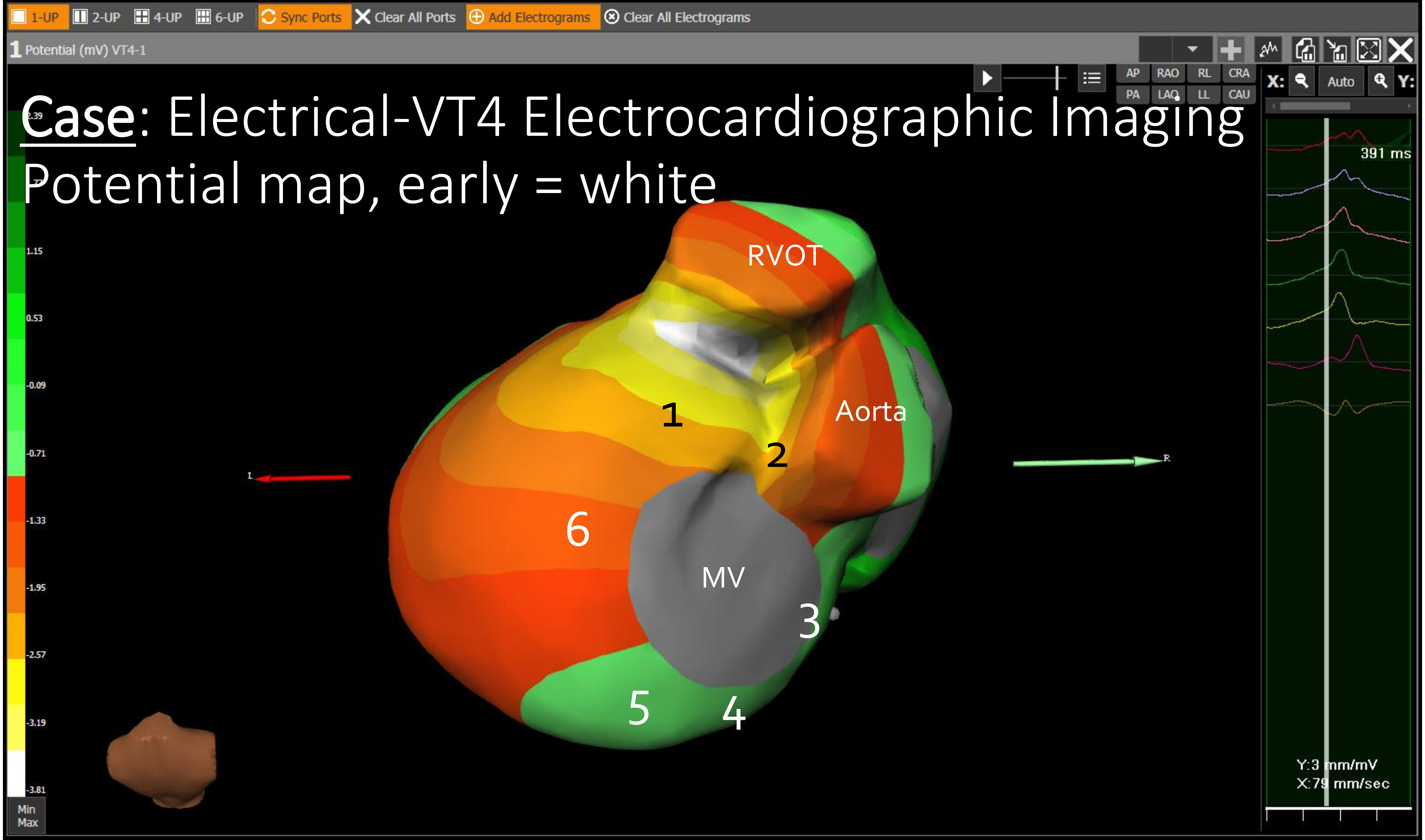

<u>Case:</u> Scar Map-Myocardial Resonance (MRI)

Basal segments: aorta, aortic valve, LV outflow, mitral valve

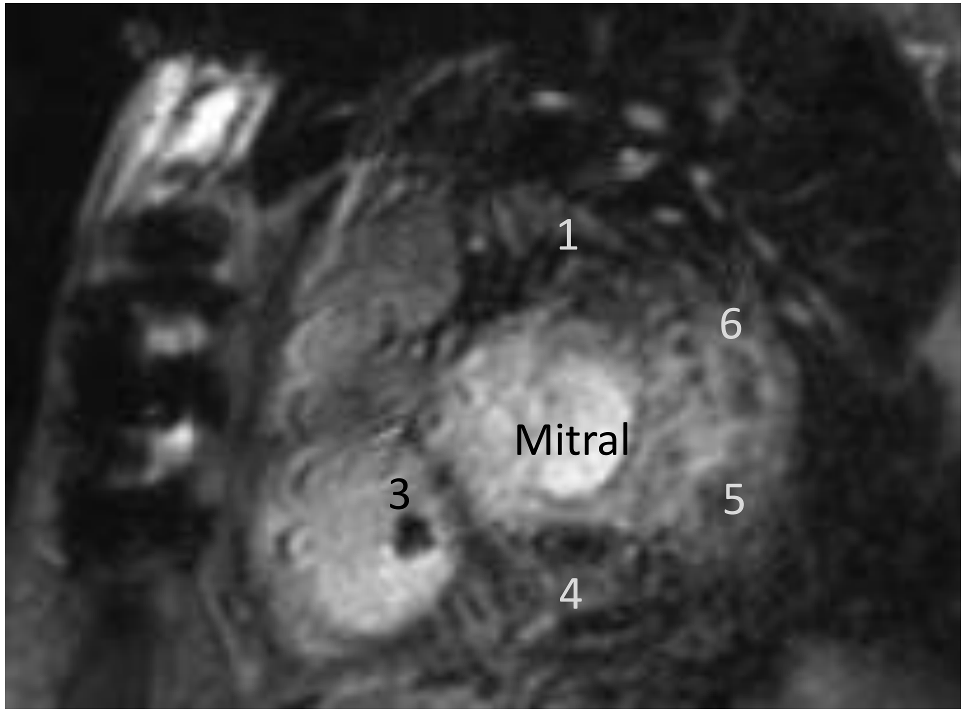

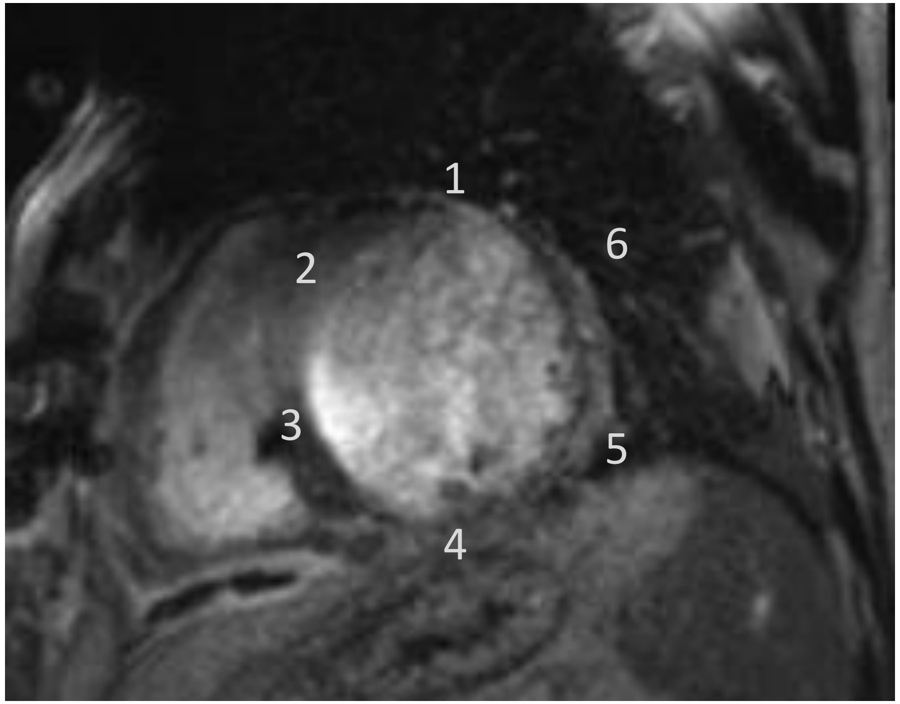

Gadolinium enhancement
****Segments 1,2,4,5,6****

Not interpretable:

Case: Scar Map-Myocardial Resonance (MRI)

Mid segments: papillary muscles

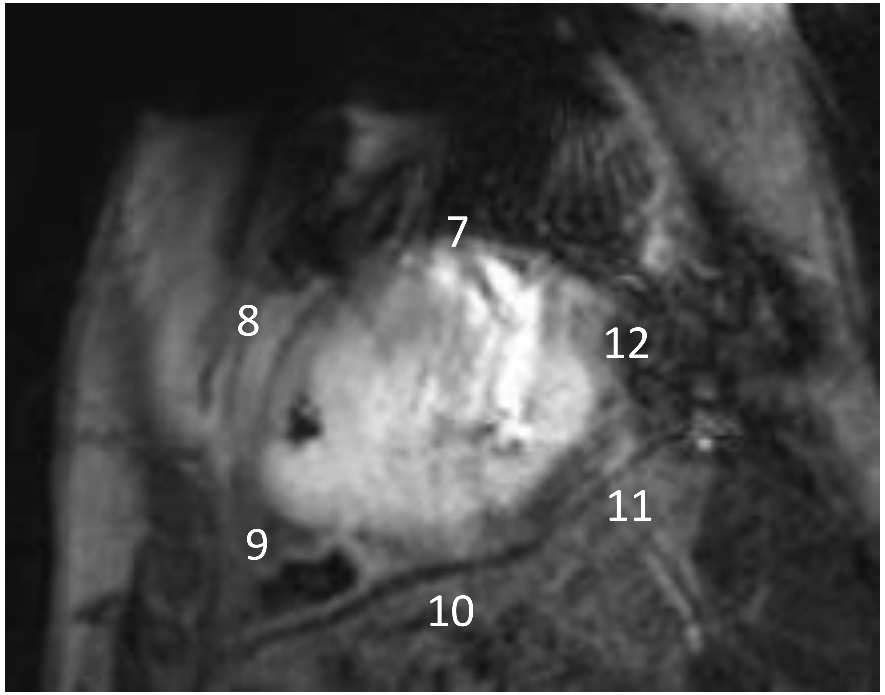

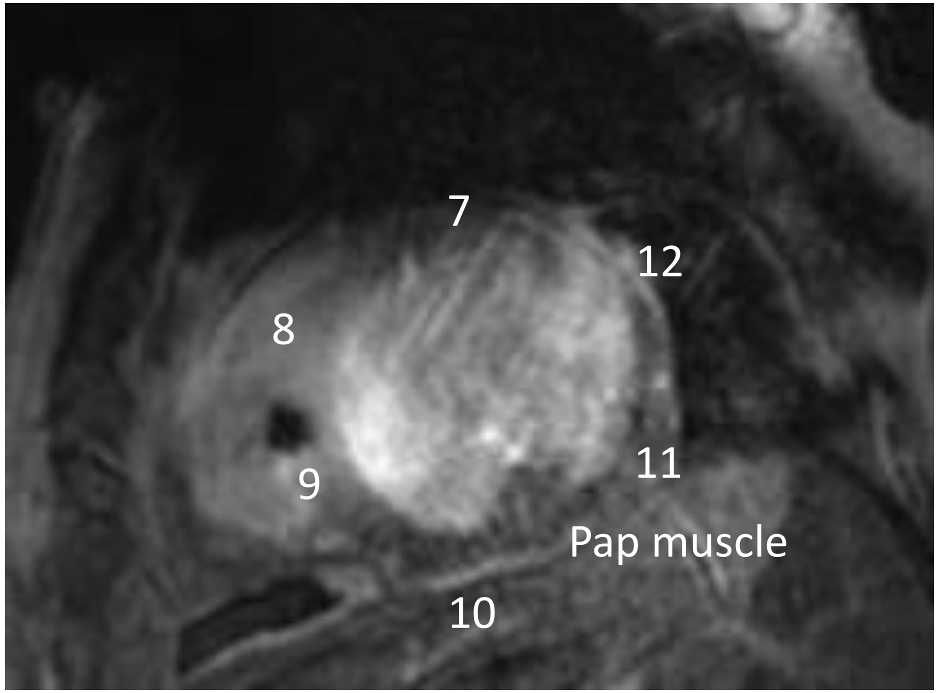

Gadolinium enhancement
Segments 8,12

Not interpretable:
Segment 7

Case: Scar Map-Myocardial Resonance (MRI)

Apical segments: no papillary muscle

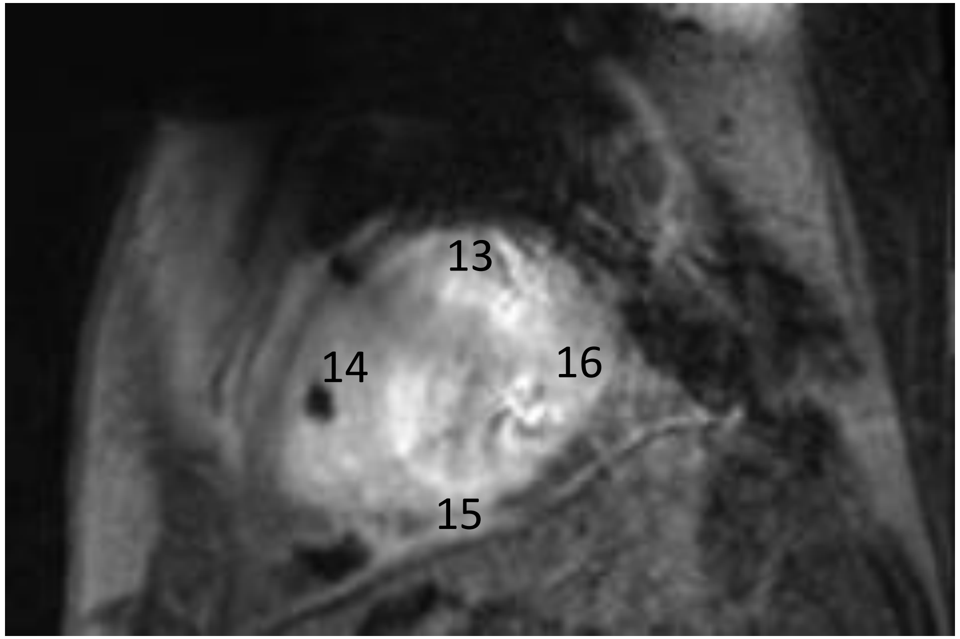

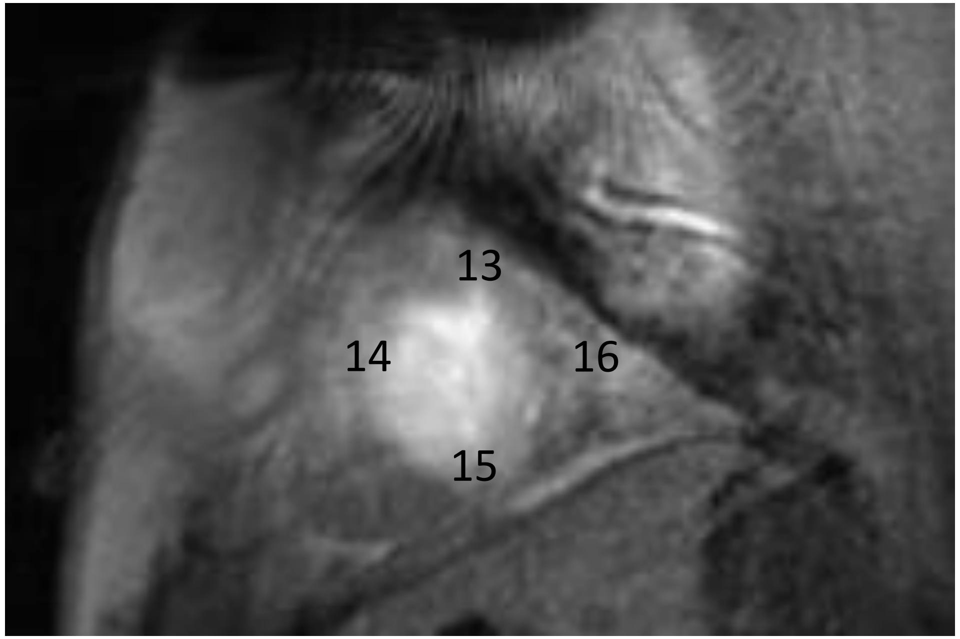

Gadolinium enhancement
****None****

Not interpretable:
13

<u>Case:</u> Scar Map-Myocardial Resonance (MRI), long axis

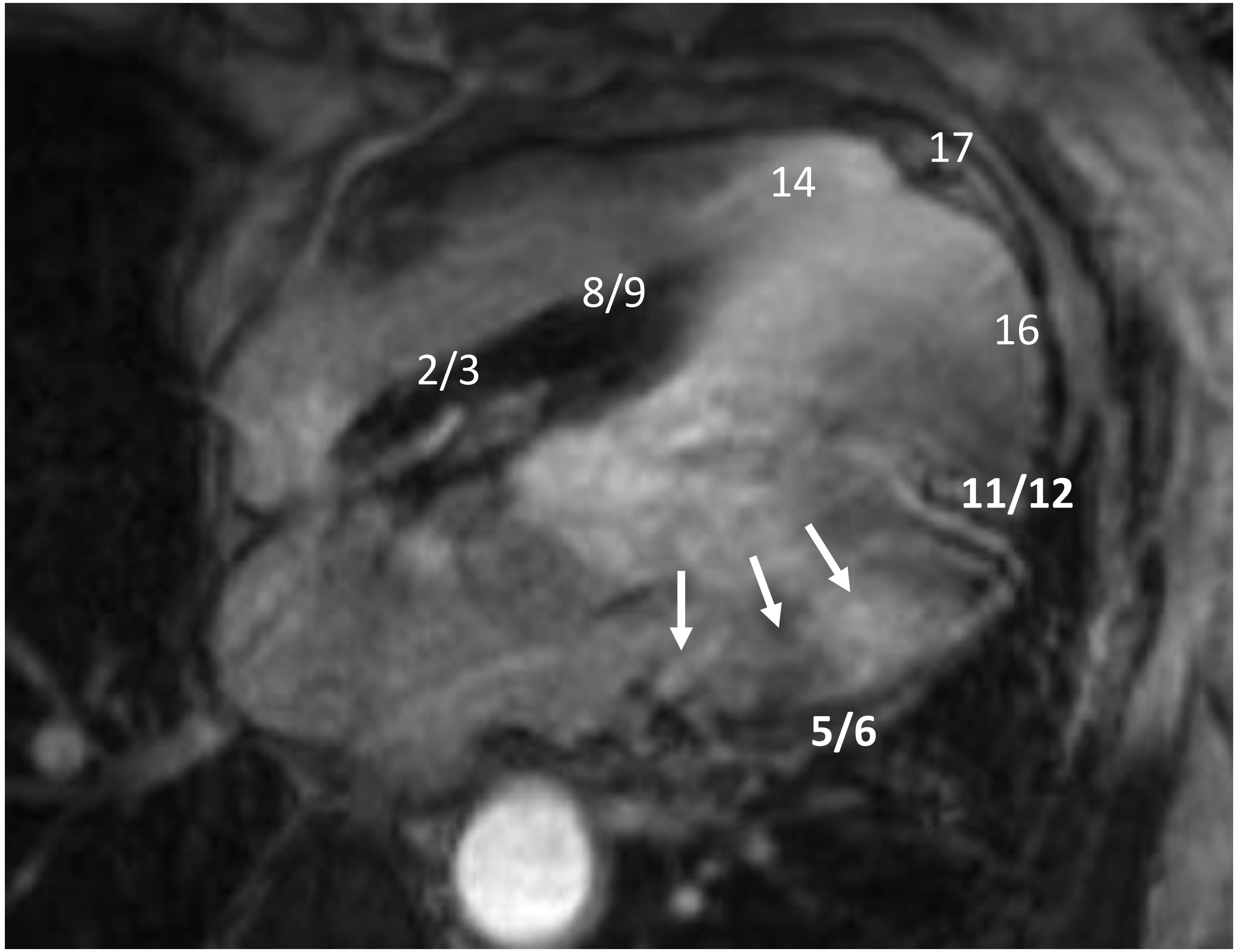

Gadolinium enhancement
****Segments 2/3, 5/6?****

Not interpretable:
Segments 11/12,14,16,17

<u>Case:</u> Scar Map – PET Inflammation

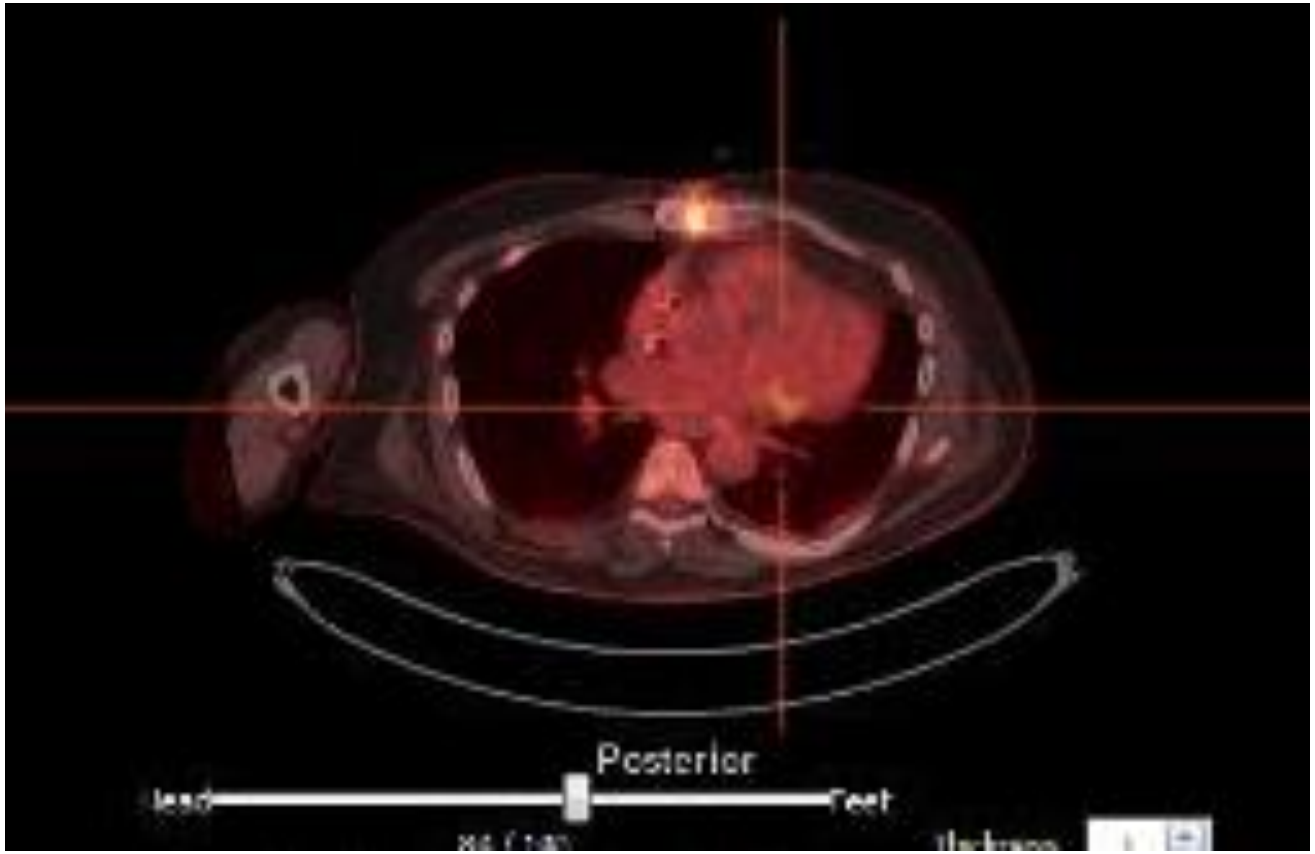

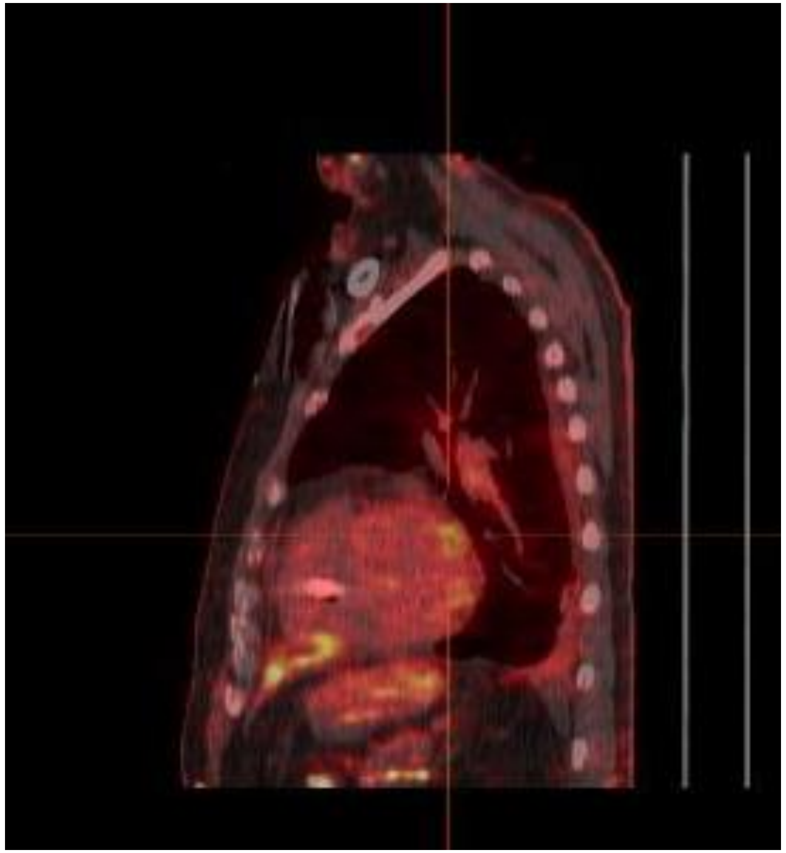

Inflammation
Segment 6

Case: Scar Map – Echocardiogram

Contrast Agent: 0.4 ml Optison Administered, (2.6 ml wasted). **Cont. Adm By:** Donna Rieger, RN

MV Structure: Normal **Motion:** Normal **Mitral Annulus:** Normal
AV Structure: tricuspid and is Normal **Motion:** Normal
Aortic root: Normal **TV:** Normal **PV:** Normal
Valvular Vegetations: none seen **Mass/Thrombi:** none seen

Measurements Normal Values from ASE-JASE 2015; 281:1-39 RA : Normal

M-Mode	(Norm.)	*2D Linear*	(Norm.)	*2D Volume*	(Norm.)	INDEXED	(Ind. Norm.)	*3D Volume*		(Indexed)
AO Root:	<3.8	AO Root: 3.4 cm	<4.0	RA: 34 ml		16.4 ml/M2	11 - 39			
LA:	<4.0	AO (Ind.): 1.6 cm/M2	<2.0	LA: 57 ml		27.6 ml/M2	16 - 34			
RV:	<2.8	LA:	<4.0	RV:			<12.7			
LV (ED):	<5.7	RV: 3.7 cm	<4.2							
LV (ES):	Variable	LV (ED): 6.5 cm	<5.9	LV (ED): 228 ml	62-150	110.2 ml/M2	<75	LV (ED):	mL/m2	<75
		LV (ES): 5.5 cm	<4.0	LV (ES): 156 ml	21-61	75.4 ml/M2	<32	LV (ES):	mL/m2	<32

LV EF: 32 % (Mod. Simpson's) (Norm. ≥ 52 %) Wall Thickness (Septum/Post. wall): 0.9 / 0.6 cm Norm. : <1.1 cm

Wall Motion Scoring: LAD RCA LCX **1=Normal, 2=Hypo, 3=Akinetic, 4=Dyskin/Aneurysm, 0=Not visualized**

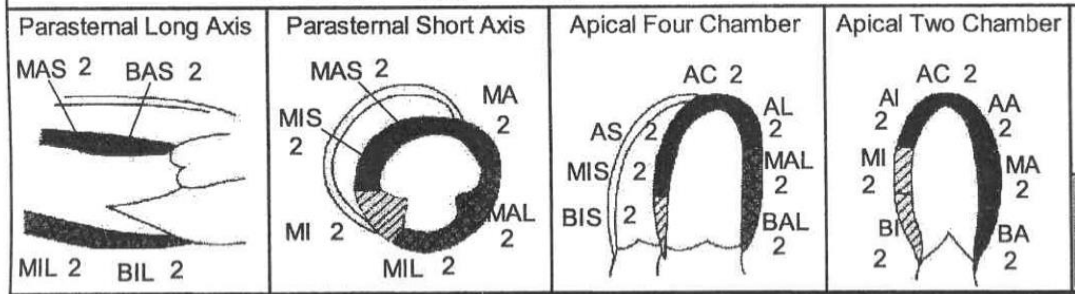

KEY:
AA - Apical Anterior
AC - Apical Cap
AI - Apical Inferior
AL - Apical Lateral
AS - Apical Septum
BA - Basal Anterior
BAL - Basal Ant. Lateral
BAS - Basal Ant. Septum
BIL - Basal Inf. lateral
BI - Basal Inferior
BIS - Basal Inf. Septum
MA - Mid Anterior
MAL - Mid Ant. Lateral
MAS - Mid Ant. Septum
MI - Mid Inferior
MIL - Mid Inf. Lateral
MIS - Mid Inf. Septum

CONTRAST ENHANCEMENT WAS EMPLOYED after initial imaging due to sub-optimal quality related to co-morbidity defined by patient's body habitus.

LV Ejection Fraction: Moderate Global reduction in LV Ejection Fraction (EF=30-40%); EF via modified Simpson's.

LV Global Longitudinal Strain: -6.8% (Normal LV Long. Strain < -19%) **RV Global Longitudinal Strain:**
RV Function: Normal **Septal Motion:** Normal
Pericardial Effusion: none seen **Atrial Septum:** Normal

Wall Motion Abnormalities
****Nonspecific****
